# Ror2 Enhances Polarity and Directional Migration of Primordial Germ Cells

**DOI:** 10.1371/journal.pgen.1002428

**Published:** 2011-12-22

**Authors:** Diana J. Laird, Svetlana Altshuler-Keylin, Michael D. Kissner, Xin Zhou, Kathryn V. Anderson

**Affiliations:** 1 Ob/Gyn Department, Center for Reproductive Sciences, University of California San Francisco, San Francisco, California, United States of America; 2 Eli and Edythe Broad Center of Regeneration Medicine and Stem Cell Research, University of California San Francisco, San Francisco, California, United States of America; 3 Developmental Biology Program, Sloan Kettering Institute, New York, New York, United States of America; 4 Biochemistry and Structural Biology, Cell and Developmental Biology, and Molecular Biology Program, Weill Graduate School of Medical Sciences, Cornell University, New York, New York, United States of America; University of Sydney, Australia

## Abstract

The trafficking of primordial germ cells (PGCs) across multiple embryonic structures to the nascent gonads ensures the transmission of genetic information to the next generation through the gametes, yet our understanding of the mechanisms underlying PGC migration remains incomplete. Here we identify a role for the receptor tyrosine kinase-like protein Ror2 in PGC development. In a *Ror2* mouse mutant we isolated in a genetic screen, PGC migration and survival are dysregulated, resulting in a diminished number of PGCs in the embryonic gonad. A similar phenotype in *Wnt5a* mutants suggests that Wnt5a acts as a ligand to Ror2 in PGCs, although we do not find evidence that WNT5A functions as a PGC chemoattractant. We show that cultured PGCs undergo polarization, elongation, and reorientation in response to the chemotactic factor SCF (secreted KitL), whereas *Ror2* PGCs are deficient in these SCF-induced responses. In the embryo, migratory PGCs exhibit a similar elongated geometry, whereas their counterparts in *Ror2* mutants are round. The protein distribution of ROR2 within PGCs is asymmetric, both in vitro and in vivo; however, this asymmetry is lost in *Ror2* mutants. Together these results indicate that Ror2 acts autonomously to permit the polarized response of PGCs to KitL. We propose a model by which Wnt5a potentiates PGC chemotaxis toward secreted KitL by redistribution of Ror2 within the cell.

## Author Summary

Egg and sperm derive from precursors in the early embryo called primordial germ cells (PGCs). The mechanisms underlying the migration of PGCs through the embryo to the forming gonads remain unclear. In a genetic screen, we identified a role for the receptor Ror2 and its ligand Wnt5a in promoting PGC colonization of the embryonic gonads. By ex vivo culture, we show that Ror2 acts autonomously in PGCs to enhance their polarized response to the chemotactic factor SCF. Asymmetric distribution of ROR2 within PGCs in vitro and in vivo suggests that signaling via Ror2 locally amplifies cell polarity in response to other directional cues. These studies identify a novel relationship between Ror2 and cKit signaling in polarized migration.

## Introduction

Primordial germ cells (PGCs) are embryonic precursors of the gametes that arise before other major cell lineages in most multicellular animals [1]. This early specification necessitates a lengthy migration through the developing embryo in order to reach the nascent ovaries or testes. In mice, epiblast-derived cells seal their germline commitment at the embryo periphery ∼e7.25, then enter the forming endoderm and travel through the elongating hindgut epithelium. PGCs make a coordinated exodus into the surrounding mesentery at e9.5 and then converge on the gonadal ridges between e10.5 and e11.5. Though exquisitely coordinated, this process is also imperfect; by e12 when migration is over, stragglers consistently remain outside the gonad in midline tissues, and are eliminated by apoptosis [2]. The importance of balanced regulation of PGC survival and migration is evident by the consequences of dysregulation: failure to survive or reach the gonad can lead to sterility, whereas inappropriate survival can lead to germ cell tumors [3], [4]. The molecular mechanisms underlying the migration of these evolutionarily essential but relatively inaccessible cells remain largely unknown in the mammalian germline. Here we conducted a forward genetic screen for germ cell defects in mouse embryos and identified an allele of *Ror2*.

Ror2 is a highly conserved receptor tyrosine kinase with homologs in many metazoans from *Aplysia* to *Drosophila* to humans [5]. Widely expressed during development, Ror2 has been implicated in chondrocyte differentiation, cochlear, craniofacial, heart, limb and gut morphogenesis in mice and humans [6]–[9]. Work in a number of different organisms suggests that Ror2 signaling affects cell polarity. In the developing mouse gut epithelium, the protein exhibits apicobasal polarity in its distribution [10]. Polarity is requisite for cells undergoing directed migration, cell division in a particular orientation, as in asymmetric divisions, and for the organization or shape of cells with respect to their neighbors, for example in convergent extension. Defects in cell shape and convergent extension have been reported in the mouse gut, organ of Corti, and Xenopus gastrula as a result of Ror2 signaling loss [9], [11]–[13]. Ror2-mediated polarized cell division has been reported in *C. elegans* [14]. A role for Ror2 signaling in directional migration has been reported in the mammalian palate [15] and in several cell lines, via c-Jun N-terminal Kinase and the actin-binding protein FilaminA [16]–[18].

Phenotypic resemblance between mouse embryos with targeted deletions of *Ror2* and those deficient for *Wnt5a* first suggested that these genes share a common pathway [6], [8], [17], [19]. Biochemical approaches later confirmed ligand-receptor interactions between Wnt5a and Ror2 via the cysteine-rich (frizzled-like) extracellular domain of Ror2 [17]. Indeed, the expression patterns of *Wnt5a* and *Ror2* virtually overlap in the primitive streak, tail mesoderm and limb buds of midgestation mouse embryos [19]–[21]. Wnt5a was similarly invoked in aspects of cell polarity, including orienftation of cell division in the limb [22], convergent extension movements and cell shape in the Xenopus gastrula [23], [24], and polarized migration in a melanoma cell line [25], [26]. Many of these different Wnt5a-Ror2 pathway mutants exhibit similarly altered distribution of polarity mediators, such as Disheveled [23], [27], [28], the Dlg-Lgl complex [24], [29], Van Gogh [29], or adhesion receptor complexes [26].

The identification of the *Ror2^Y324C^* mutant in an unbiased screen for PGC defects brings to light a previously unrecognized function of Ror2 in germ cell development. We show here that Ror2 and its putative ligand Wnt5a promote efficient migration of PGCs to the embryonic gonads. These studies demonstrate a cell intrinsic function for Ror2 in potentiating the polarized response to secreted KitL, drawing a new link between Ror2 and Kit signaling in PGC migration.

## Results

### An ENU allele of Ror2 and its expression in PGCs

**Figure 1:**
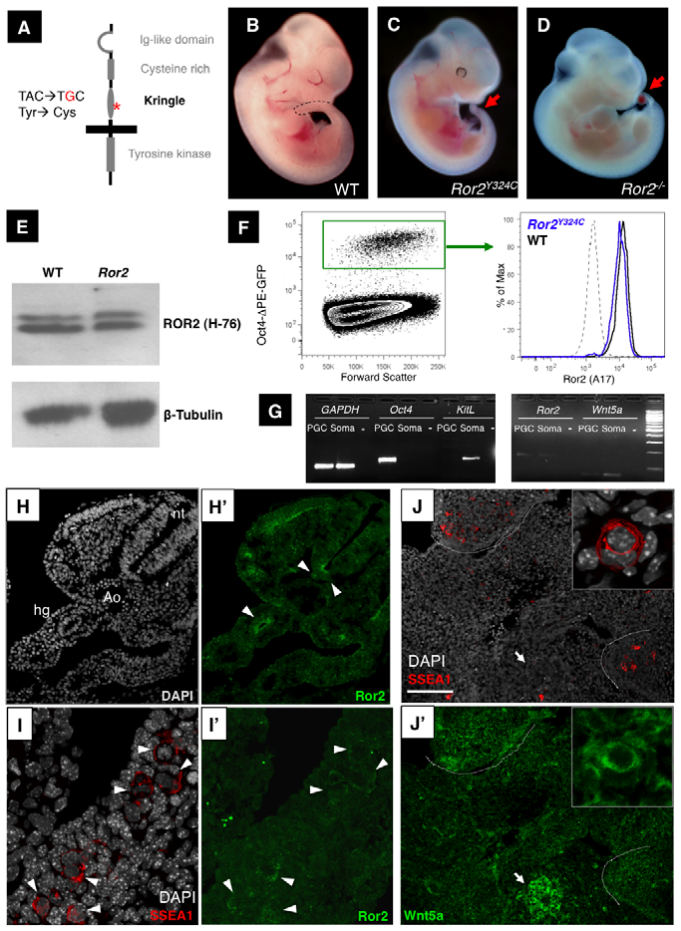
A *Ror2* ENU allele and expression in PGCs. (A) Schematic of the Ror2 gene product with indicated mutation at nucleotide 1203 and predicted amino acid change. (B–D) *Ror2^Y324C^* homozygotes and (C) *Ror2* null embryos (D) at ∼e10.75 exhibit a short tail (arrow). (E) Whole embryo lysates from e9.5 immunoblotted for ROR2 and β-Tubulin indicate that protein is present in *Ror2^Y324C^* homozygous mutants. (F) PGCs at e11.5 flow cytometrically identified by the Oct4ΔPE-GFP transgene (left) show comparable levels of ROR2 intracellular staining (right). (G) RT-PCR from WT sorted Oct4ΔPE-GFP^+^ (denoted PGC) and GFP^neg^ tail somatic (soma) cells, and no cDNA controls (-) at e10.0 show the presence of *Ror2* and *Wnt5a* in both populations. The purity of Oct4ΔPE-GFP^+^ cells was confirmed by the presence of *Oct4* and absence of *KitL*. Both images are from the same gel, with 100 bp ladder shown at right. (H–H′) ROR2 immunostaining (green) in the ventral neural tube, hindgut and somites (arrowheads) of WT e10.5 sections. Scale bar = 100 um. (I–I′) ROR2 immunostaining (green) was present throughout WT e10.5 dorsal mesentery and appeared enriched on the surface of PGCs, coincident with SSEA1 (red). Scale bar = 24 um. (J–J′) In e11.5 transverse sections, WNT5A immunostaining (green) was enriched in the intestine (arrow) and gonadal ridges (dashed lines) where the majority of PGCs (red) reside. Nuclei are shown in grey. Variable levels of WNT5A signal were observed in PGCs, such as these two adjacent examples (inset, 5× magnification). Scale bar = 118 um.

As an unbiased approach to identifying new genes involved in mouse germ cell development, we conducted a genome-wide recessive ethylnitrosourea (ENU) mutagenesis screen for PGC defects in e9.5 embryos [30]. One of the mutations identified based on the presence of ectopic PGCs mapped to the region of *Ror2*. An A to G transition in exon 7 at nucleotide 1203 causes a tyrosine to cysteine substitution at position 324 (Y324C) of the ROR2 predicted protein (Figure 1A). This missense mutation falls in the kringle domain, a conserved structural motif in the ROR2 extracellular domain. *Ror2^Y324C^* homozygous embryos exhibit defects in tail elongation (Figure 1B, 1C) and somite segmentation, similar to the *Ror2* targeted deletion allele (Figure 1D) [8], [17]; like the knockout, *Ror2^Y324C^* mutants die perinatally. Ror2 immunoblotting on e10.5 embryo lysates revealed a double band at approximately 200 kD; both bands were present in similar amounts between WT and *Ror2^Y324C^* mutants (Figure 1E). In humans, missense mutations in the *hRor2* cysteine rich, kringle and tyrosine kinase domains that are associated with Robinow syndrome cause the protein to be retained in the endoplasmic reticulum [31]. We examined the expression of ROR2 at e11.5 by intracellular staining with an antibody directed against the cytoplasmic tail of the receptor; by flow cytometry signal was present at similar levels in WT and *Ror2^Y324C^* (Figure 1F, right). These experiments suggest that the mutation does not affect protein stability but do not discriminate between its normal or abnormal subcellular localization.

To determine whether Ror2 is expressed in PGCs, we employed a transgenic mouse strain, *Oct4ΔPE-EGFP*, which expresses Enhanced Green Fluorescent Protein (GFP) under a modified Oct4 reporter that is specific to PGCs during mid-gestation [10], [32] (Figure 1F). By flow cytometry, ROR2 intracellular staining was present within the GFP^+^ population at e11.5 (Figure 1F). Furthermore, when Oct4ΔPE-EGFP^+^ PGCs were purified flow cytometrically, *Ror2* transcript could be detected by semi-quantitative RT-PCR; more transcript appeared to be present in GFP^negative^ cells from embryo tails (denoted ‘soma’; Figure 1G), where high levels of *Ror2* have been previously detected by in situ hybridization [8]. The purity of sorted PGCs was confirmed by RT-PCR for *Oct4*, which was absent in somatic cells, and *KitL*, which was confined to soma (Figure 1G). ROR2 protein was similarly detected in histologic sections with two different antibodies; signal appeared to be concentrated at the apical surface of the hindgut and somites [33] and in the ventral neural tube in wild type embryos (Figure 1H–1H′), as previously reported [34]. ROR2 was also present throughout the e10.5 dorsal mesentery and enriched at the membrane of wild type PGCs (Figure 1I, 1I′). These studies confirm the expression of Ror2 mRNA and protein in migratory and postmigratory PGCs, as suggested by previous microarray data [35], and demonstrate the stable expression of Ror2^Y324C^ mutant protein.

A major ligand for Ror2 is believed to be Wnt5a. *Wnt5a* mRNA expression in the tail and hindgut of the embryo overlaps that of *Ror2*, although precisely which cells secrete Wnt5a remains unclear [19]–[21]. By RT-PCR we determined that *Wnt5a* transcript is present in sorted Oct4ΔPE-EGFP^+^ PGCs, although it is more abundant in GFP^negative^ somatic cells of the tail and hindgut (Figure 1G). In histological sections stained with a WNT5A antibody, we observed bright foci as well as intercellular signal in the intestine and gonadal ridges (Figure 1J–1J′), which both lie on the PGC migratory route. Upon closer examination, WNT5A could be detected at variable levels at or near the surface of PGCs (Figure 1J, inset). These results collectively identify a role for Ror2 in PGC development and raise the possibility that PGCs perceive paracrine or autocrine WNT5A signals via the Ror2 receptor.

### PGCs are gradually depleted in *Ror2* and *Wnt5a* mutants

**Figure 2:**
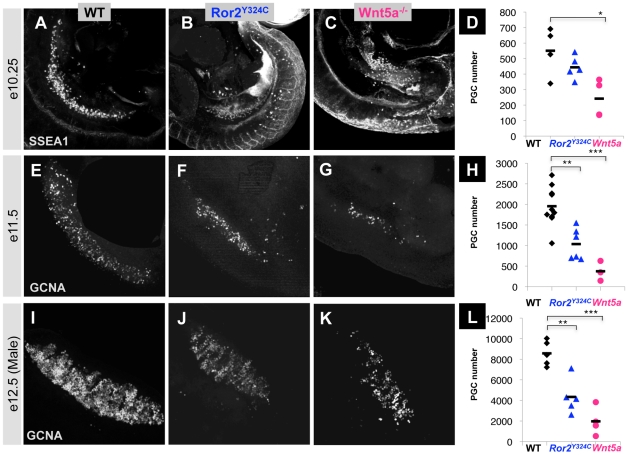
PGC depletion in *Ror2* and *Wnt5a* mutants. (A–C) PGCs were visualized by wholemount SSEA1 immunostaining with SSEA1 antibody in e10.25 WT, *Ror2*, and *Wnt5a* embryos. (E–G) Gonadal ridges from e11.5 stained with GCNA, and (I–K) e12.5 male gonads stained with GCNA antibody. The caudal end is down in all images. (D, H, L) Quantification of PGCs in the entire e10.25 embryo, e11.5 and e12.5 gonads from confocal stacks, with individuals denoted as WT (diamond), *Ror2^Y324C^* mutants (triangle), and *Wnt5a* null (circle), and means indicated as bars. Consistent with appearances, a significant reduction of PGCs was observed in *Wnt5a* and from e11.5 onward in *Ror2^Y324C^*. We noted no significant difference in the number of PGCs between XX and XY gonads at e12.5 (not shown). Results of the Student's t-test are indicated, * p<0.05, **, p<0.01, ***p<0.001.

We next characterized the phenotypes of PGCs in *Ror2^Y324C^* mutants. In e10.5 embryos stained with SSEA1 antibody [36], [37], PGCs can be visualized migrating through the dorsal mesentery (Figure 2A). In *Ror2^Y324C^* mutants, PGCs do not migrate rostrally, but remain in the mesentery surrounding the caudal hindgut (Figure 2B), as well as on the surface of the tail and in the allantois (Figure S3B, S3C). At e11.5, immunostaining with GCNA (a marker of postmigratory PGCs [38]) revealed a reduction in the number of PGCs within *Ror2^Y324C^* gonad primordia compared to wild type; furthermore, the distribution of *Ror2^Y324C^* PGCs was skewed toward the caudal end of the gonad and extragonadal PGCs were increased in midline tissues (Figure 2E, 2F). At e12.5, male and female *Ror2^Y324C^* gonads appeared less densely populated with PGCs (Figure 2I, 2J, female not shown).

We developed techniques for the quantification of PGCs in the entire embryo or embryonic gonad with confocal imaging and 3D analysis (see Methods). The mean number ± standard deviation of PGCs in mutants at e10.25 (443±73) was similar to wild type (551±157; Figure 2D), in spite of their abnormal distribution. However, at e11.5, the number of *Ror2^Y324C^* PGCs in gonads was diminished (1243±369) compared to wild type (2598±265, p = 0.0002; Figure 2H). At e12.5 this difference persisted (p = 0.009), as 7058±2282 PGCs were counted in wild type gonads and 3825±1144 in *Ror2^Y324C^* (Figure 2L); male and female were combined here, as their numbers were similar. The PGC estimates and corresponding doubling time found in wild type embryos (13.4–16.7 hours) are similar to those reported previously [39]. The doubling time for *Ror2^Y324C^* PGCs falls within this range for postmigratory PGCs, but was more protracted from e10.25–11.5 (20 hours), predicting an earlier decline in proliferation or rise in apoptosis. We compared the phenotype of *Ror2^Y324C^* PGCs to that of a targeted *Ror2* knockout allele [8]. At e11 and e12, we observed a similar PGC decrease compared to age-matched wild type C57Bl/6 littermates (Figure S1). Despite genetic background differences, the PGC deficit in *Ror2^−/−^* embryos was indistinguishable from that resulting from our point mutation. This similarity suggests that *Ror2^Y324C^* is a strong loss of function allele.

Previous work has shown that Ror2 lies downstream of Wnt5a both biochemically and genetically [17], [19], [40]. Therefore, we examined the PGCs of *Wnt5a* null mutants and found a more pronounced and earlier deficit compared to *Ror2*. At e10.5, *Wnt5a^−/−^* PGCs were similarly caudally distributed (Figure 2C) but were already depleted in number (242±121) compared to wild type (Figure 2D). We noted significant reductions at e11.5, when 310±148 PGCs were present in *Wnt5a* gonads (Figure 2G, 2H), and by e12.5 this number increased to 1587±985 (Figure 2K, 2L). Consistent with biochemical data [17], [40], the greater severity of the *Wnt5a* germ cell phenotype suggests that this ligand operates through other receptors besides Ror2. Together, these studies demonstrate that *Wnt5a* and *Ror2* mutants are phenocopies in the PGC compartment, which corroborates their function there as ligand and receptor.

### Increased programmed cell death of PGCs in *Ror2* and *Wnt5a* mutants

**Figure 3:**
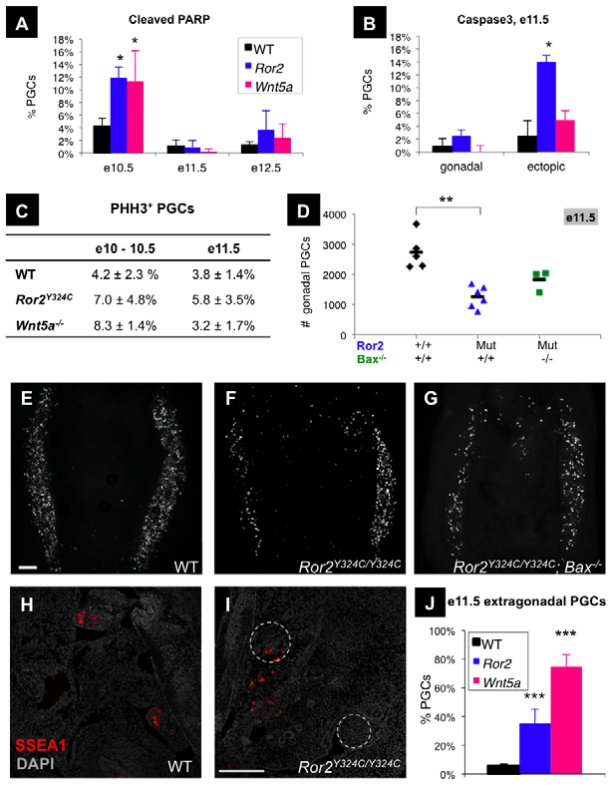
Increased PGC apoptosis and impaired colonization of the gonads in *Ror2* and *Wnt5a* mutants. (A) The frequencies of apoptotic PGCs were similar between all genotypes in e11.5 and e12.5 gonads, but increased in *Ror2* and *Wnt5a* migratory PGCs at e10.5 (n = 4 mutants). (B) When examined separately in e11.5 sections, the frequency of apoptotic PGCs was similar in the gonads, but increased among ectopic extragonadal *Ror2* PGCs compared to WT. (C) The frequencies of PHH3^+^ (ectopic and non-ectopic) PGCs determined from tissue sections at e10.5 and wholemount gonads at e11.5 did not differ between WT and either mutant by t-test (mean and SD of n≥3 embryos). (D–G) The number of PGCs within *Ror2^Y324C^* e11.5 mutant gonads was not rescued to WT levels (p = 0.065) on a *Bax* null background, quantified in (D). GCNA staining of e11.5 wholemount gonadal ridges in stage-matched WT (E) and *Ror2^Y324C^* single mutants (F) and *Bax; Ror2^Y324C^* double mutants (G). Scale bar = 120 um. (H–J) Excess ectopic PGCs were observed in e11.5 histologic sections of *Ror2^Y324C^* (I) and *Wnt5a* (not shown) stained with SSEA1 and DAPI (gonads indicated by dashed circles, scale bar = 100 um). (J) The proportion of extragonadal PGCs in *Ror2^Y324C^* and *Wnt5a* sections (mean and SE of n = 4 slides, ∼10 sections each) was significantly increased compared to WT. Results of the Student's t-test are indicated, * p<0.05, **, p<0.01, ***p<0.001.

To investigate the cellular mechanism underlying the PGC deficit in *Ror2* and *Wnt5a* mutants, we extended our quantitative imaging in the embryonic gonad to include markers of proliferation and death. We performed triple immunofluorescence for GCNA, as well as phospho-histone H3 (PHH3), and cleaved PARP to quantify subsets of mitotic and apoptotic PGCs, respectively (Figure S2). No differences were observed in cPARP expression among postmigratory PGCs in wild type, *Ror2*, or *Wnt5a* gonads (Figure 3). However, analysis of e10.5 embryo sections revealed an increase in cPARP expression among still migratory PGCs in *Ror2^Y324C^* (11.9±1.6%) and *Wnt5a* (11.4±4.8%) relative to wild type (4.3±1.3%; Figure 3A, staining shown in Figure S3). This discrete wave of apoptosis preceded any observed loss in cell number in *Ror2* mutants. We next compared the frequencies of programmed cell death between PGCs within and outside the e11.5 gonad. Caspase3 staining in histologic sections revealed similar frequencies in properly localized PGCs, but increased apoptosis among extragonadal *Ror2^Y324C^* germ cells (14.0±1%) compared to wild type (2.4±2.4%) and *Wnt5a* (4.9±1.2%; Figure 3B). Examination of PGC proliferation by PHH3 staining did not reveal significant differences in the frequency of proliferating PGCs between wild-type, *Wnt5a*, or *Ror2^Y324C^* embryos at e10.25 or e11.5 (Figure 3C); despite several hundred PGCs counted in each genotype at each stage, the variation was large. Together these results demonstrate that increased apoptosis rather than reduced proliferation contributes to the PGC deficit in *Ror2* and *Wnt5a* mutants.

### Ror2 is required for efficient PGC colonization of the gonads

Restriction of the observed burst of programmed cell death to migratory PGCs, together with its absence in gonadal PGCs, suggested that the location of mutant germ cells could be a factor in their elimination. On one hand, migrating mutant PGCs could be more sensitive to the reduced levels of survival factors such as KITL and SDF1 in the dorsal mesentery as compared to the gonad [2], [41], [42], [43], where they are more protected from death. On the other hand, inefficient migration may lead to an accumulation of ectopic *Ror2* PGCs, which die in an environment lacking survival factors [2]. To distinguish between these possibilities, we rescued PGC apoptosis in *Ror2* mutants by generating double mutants with a targeted knockout of the pro-death gene Bax. Previous work established an increase in ectopic PGCs in e11.5 *Bax* single mutants due to the lack of apoptosis of mis-migrated PGCs, although the total number of PGCs remained unchanged [2]. Genetic ablation of *Bax* in *Ror2^Y324C^* mutants increased the number of midline and ectopic PGCs, but did not restore the number of PGCs in the gonads. At e11.5, 1815±362 PGCs were counted in *Ror2; Bax* double mutant gonads, which did not differ from 1275±359 in stage matched *Ror2* littermates (p = 0.07; Figure 3D). Although *Bax* does not rescue PGCs in *Ror2* gonads, a significant increase in the total number of PGCs in the entire aorta-gonad-mesonephros region of double mutants compared to *Ror2* single mutants (p = 0.036; data not shown) reflects rescue of ectopic PGC death throughout the midline in *Ror2; Bax* (Figure 3G, compared to Figure 3E, 3F). This result suggests that defects in migration are primary to the defects in PGC survival in *Ror2* mutants.

We next directly compared the efficiency of PGC migration in mutants. When quantified in histological sections at e11.5 (Figure 3H, 3I), ectopic (extragonadal) PGCs comprised over 70% of the total PGCs in *Wnt5a* mutants, and 30% in *Ror 2^Y324C^*, compared to less than 5% in wild type (Figure 3J). Poor cell trafficking could therefore account for the loss of gonadal PGCs of both mutants at e11.5. However, it remained unclear whether morphologic differences in the caudal hindgut of both mutants cause the observed migration defects. Indeed, morphological and molecular analysis revealed a shortening and widening of the *Ror2^Y324C^* caudal hindgut at e9.5 (Figure 1E, Figure S4), which corresponds to the PGC exodus from the hindgut. Upon examining embryos before hindgut formation, we confirmed that the location and number of early PGCs were indistinguishable from wild type in *Ror 2^Y324C^* as well as *Wnt5a* at e7.5–8.0 (Figure S5). By e9.0, we observed ectopic PGCs accumulated in the allantois, throughout the tail mesoderm, and caudal hindgut of *Ror2^Y324C^* mutants (Figure S6A–S6C). However, this phenotype does not distinguish between the possibilities of an intrinsic PGC migration defect versus a structural abnormality that hindered the passage of PGCs from the allantois into the hindgut pocket. [44].

Given the previously demonstrated expression of *Wnt5a* throughout the allantois and primitive streak [19], we wondered whether it could act chemotactically to draw PGCs from the allantois into the hindgut. To address this possibility, we implanted beads coated with WNT5A into the caudal region of e8.0 embryos. Control BSA-coated beads delivered to the hindgut pocket did not disrupt embryo or PGC development over 24 h culture (Figure S6D, S6G). Beads soaked in recombinant WNT5A or concentrated conditioned medium did not alter the course of PGCs, whether placed directly in or near their path (Figure S6E, S6H). Consistent with previous reports [43], beads similarly impregnated with the known chemoattractants SDF1and Stem Cell Factor (SCF, or secreted KitL) affected migration of PGCs at close range, inducing occasional deviation from their normal route (Figure S6F, S6I). Although we did not assess biological activity of Wnt5a-soaked beads, when PGCs were explanted and cultured over 24 hours, we did measure a modest increase in their number in the presence of recombinant WNT5A, suggesting that WNT5A is biologically active (Figure S6J). Collectively these results could indicate that WNT5A may not act as a direct chemotactic cue for PGCs; rather, they suggest that Wnt5a and Ror2 could have a permissive role to allow the response of PGCs to other navigation signals.

### Ror2 autonomously enhances chemotactic response of PGCs to SCF in vitro

**Figure 4:**
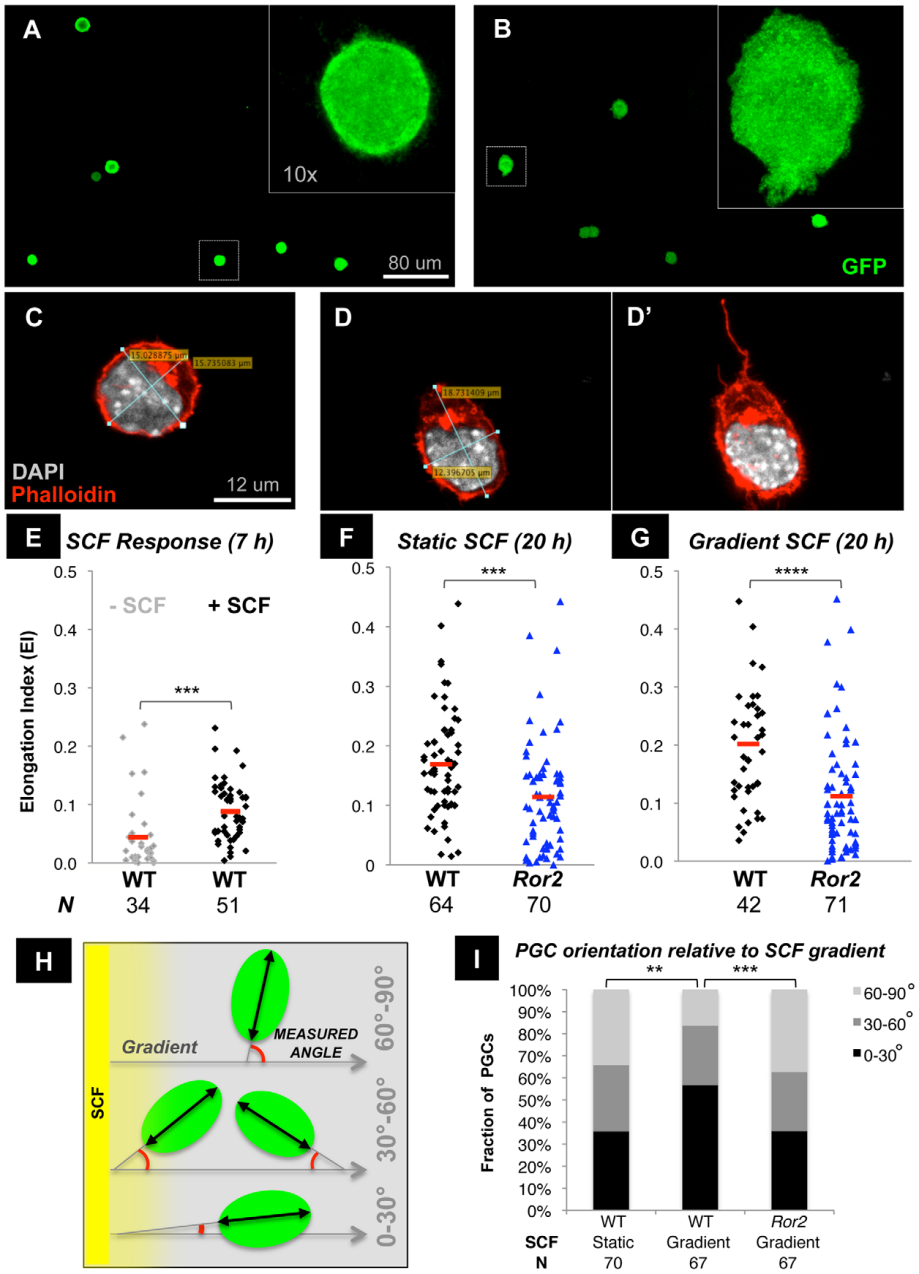
Impaired elongation and alignment with an SCF gradient in cultured *Ror2^Y324C^* PGCs. (A–B) Sorted e9.5 Oct4-ΔPE-GFP^+^ PGCs cultured 7 h on trigel without SCF (A) and with 50 ng/ml SCF (B), inset magnified 10×. (C–G) Cell axis measurements of the largest plane of the cell performed after staining with Phalloidin and DAPI are shown for representative round (C) and elongated (D) PGCs cultured ex vivo. A maximal projection of the confocal stacks (D′) depicts actin filament extensions of the elongated cell. An elongation index (EI) is calculated by (A_Long_−A_Short_)/(A_Long_+A_Short_) such that round cells approximate 0. The EI of WT PGCs increases after 7 h culture in static 50 ng/ml SCF (E), and is more pronounced by 20 h in WT (F). *Ror2* PGCs remain less elongated in static (p = 0.0005) as well as graded SCF (G, p = 0.0004). Cell axis measurements shown are from n≥3 experiments. (H–I) The angle between the long cellular axis and the gradient was measured to determine PGC alignment with respect to SCF (H). WT PGCs cultured 20 h in graded SCF are biased toward low angles, whereas *Ror2* PGCs exhibit a more random orientation, similar to WT in static SCF (I). Cell angle data represent n = 3 experiments.

Reduced PGC colonization of the gonads in *Ror2* and *Wnt5a* mutants could result from disruptions in hindgut architecture or from intrinsic defects in PGC migration. The expression of Ror2 in both PGCs and their surrounding tissues does not provide any insight. In fibroblasts, previous work showed that WNT5A induces motility, cell shape change, and chemotaxis via Ror2 [16], [45]. We did not observe PGC chemotaxis toward a WNT5A source in cultured embryos. Other work shows that WNT5A polarizes melanoma cells when a chemotactic gradient is present [26]. We sought a direct test of migratory capacity of isolated *Ror2* PGCs. However, when sorted from e9.5–10.5 embryos using the *Oct4ΔPE-EGFP* reporter, we did not observe any migration of wild type PGCs toward SDF1 or SCF in a transwell assay, as previously reported [46]. However, Farini et al. also showed that SCF elicited cytoskeletal changes and membrane protrusions in isolated PGCs over a short period [46]. We replicated this result using flow cytometrically purified *Oct4ΔPE-EGFP* ^+^ cells from e9.5 embryo posteriors and maintained on Matrigel in serum-free media. Without the support of feeder cells, which provide growth factors, survival was poor and PGCs appeared round and devoid of filopodia (Figure 4A). As reported [46], the addition of SCF induced morphological changes in PGCs, including the acquisition of membrane protrusions and ellipsoid shape (Figure 4B). We noted that the shape assumed by *Ror2* PGCs cultured in these conditions differed from wild type, and therefore endeavored to quantify this morphology. Using phalloidin to define the F-actin cytoskeleton, we measured the longest cellular axis and the orthogonal short axis of the cell body; we then computed an Elongation Index (A_Long_−A_Short_)/(A_Long_+A_Short_), which approaches zero for round cells, such as the example in Figure 4C. Elongated cells often extended filopodia or lamellopodia, which were not included in the measurement, but which usually aligned with the long axis (Figure 4D–4D′). Following 7 hours of culture without SCF, a mean Elongation Index (EI) of 0.044 was observed in wild type PGCs, which increased to 0.088 in the presence of SCF (p = 0.0005; Figure 4E). PGC elongation continued to increase in culture up to 20 hours in SCF, to a mean EI of 0.169, (Figure 4F). By contrast, *Ror2* PGCs mutants cultured in parallel exhibited a mean EI of 0.114 in SCF, which is significantly lower than mixed wild type and heterozygous PGCs (p = 0.005). When SCF was excluded from the media, but a strip of Matrigel was introduced along one side of the culture well to produce a gradient, the elongation response of WT PGCs was similar to that in static SCF, with a mean EI of 0.20; the graded source of SCF did not increase the EI of *Ror2* PGCs: mean EI of 0.11, p = 0.0004 (Figure 4G). Short axis dimensions did not differ between wild-type and mutant PGCs (data not shown), but as we did not assess the z-axis length, these results do not exclude the possibility that *Ror2* PGCs occupy less volume instead of remaining more spherical than wild type following SCF treatment.

We also examined the capacity of PGCs to align with a chemotactic gradient. Using the long cellular axis explained above, we measured the angle between this axis and a line orthogonal to the source of SCF (schematized in Figure 4H). When SCF was uniformly present in the media (here termed static), the orientation of WT PGCs was randomly distributed between 0 and 90° from an arbitrary line, as would be expected. However, following 20 hours in an SCF gradient, wild-type PGC orientations were biased toward lower angles; that is, they showed greater alignment parallel to the gradient (Figure 4I, p = 0.0018). *Ror2* PGCs did not preferentially orient toward the SCF source, but were randomly distributed in their orientations (Figure 4I, p = 0.0004). Taken together, these in vitro studies reveal a compromised ability of *Ror2* PGCs to respond to SCF, either by elongating or orienting toward a chemotactic gradient. Because these assays were carried out in the absence of feeder cells using Oct4-ΔPE-EGFP^+^ cells sorted to >95% purity, the observed defects must be cell-intrinsic.

### Ror2 promotes coordinated PGC elongation and polarized positioning of the Golgi apparatus

**Figure 5:**
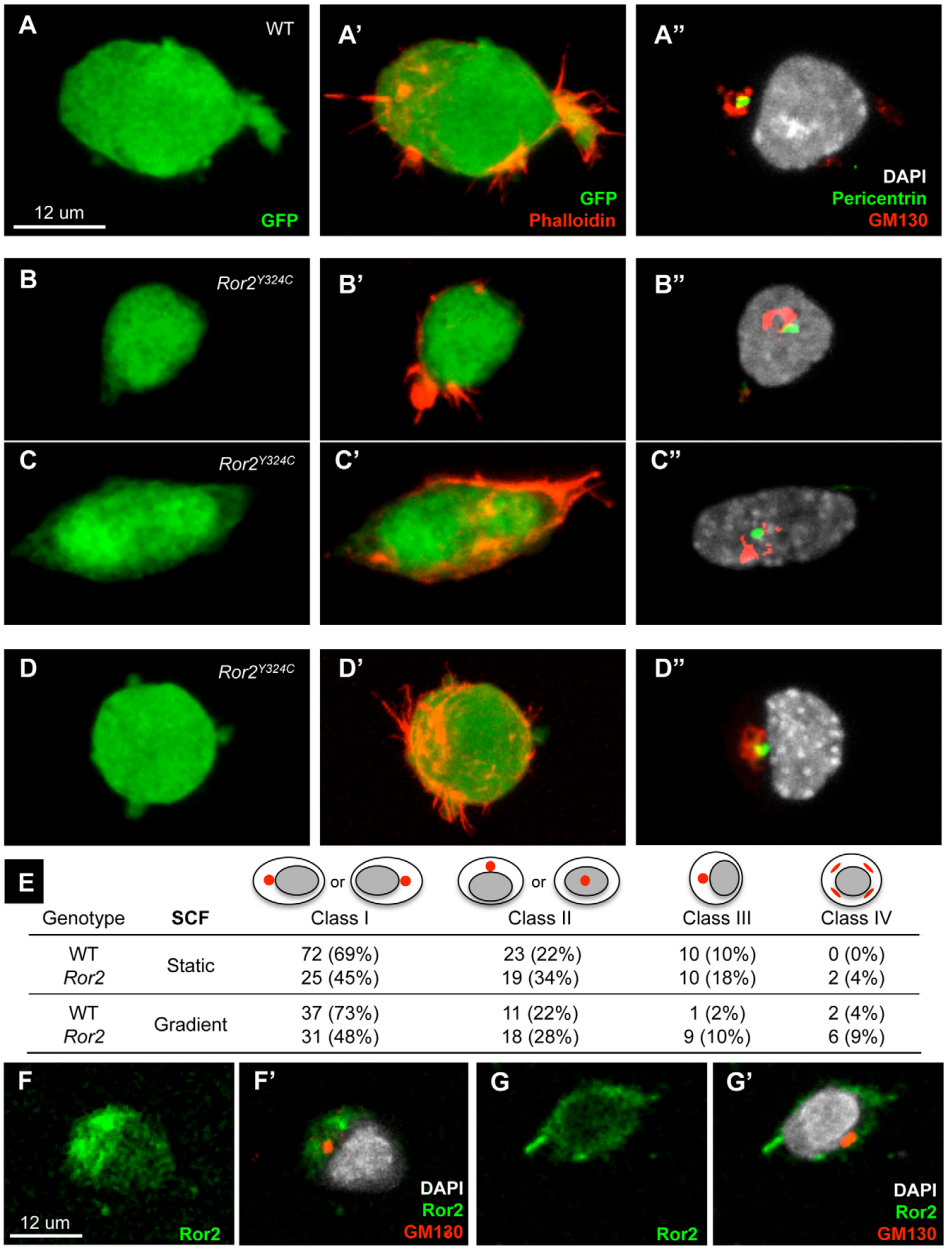
Reduced polarization by SCF in cultured *Ror2^Y324C^* PGCs. (A–D) Actin, centrosome and Golgi positions were examined in 20 h ex vivo cultured Oct4-ΔPE-GFP^+^ PGCs in the presence of SCF by GFP, Phalloidin, Pericentrin and GM130 immunofluorescence. (A–A″) An example of asymmetrically distributed GM130 and Pericentrin of an elongated cell, categorized as Class I. Class II contains elongated cells with centrally located GM130 and Pericentrin (B–B″, C–C″). Geometrically round cells comprise Class III, with GM130 and Pericentrin adjacent to the nucleus (D–D″). Dispersed GM130 was observed in a small fraction of cells that are likely dividing (not shown). (E) Quantification of cultured PGCs reveals a significant reduction of elongated *Ror2* PGCs exhibiting asymmetric GM130/Pericentrin distribution (Class I, p = 0.005), similarly in static or graded SCF by Fisher's exact test. Also, in static SCF the number of elongated *Ror2* PGCs with geometrically central GM130/Pericentrin distribution (Class II) was significantly higher than in WT (p = 0.029). Counts were accumulated from n = 3 experiments. (F–G) Ror2 and GM130 immunofluorescence on WT PGCs similarly cultured in SCF shows ROR2 localization on filopodia (F–F′) and asymmetrically on the cell surface and cytoplasm (G–G′).

Polarized cell migration depends upon the perception of an extracellular chemotactic gradient, the acquisition of polarized molecular or membrane components, and ensuing changes in cellular organization, including cytoskeletal elements and organelles [47]. We observed an overall reduction in *Ror2* PGC shape change and alignment in the presence of an SCF gradient compared to wild type. This phenotype could result from the impaired perception of a chemotactic cue or diminished capacity to respond. As little is known about PGC polarity, we first examined the localization of two subcellular structures involved in polarized responses of migratory cells, the Golgi apparatus and the centrosome; identified here by GM130 (Golgi) and Pericentrin (centrosome) immunofluorescence, these organelles are positioned by microtubules in response to polarity cues [48]. Following SCF exposure, cultured wild type and *Ror2* PGCs both elaborated F-actin-rich extensions (Figure 5A′, 5B′, 5C′). GM130 and Pericentrin staining was observed colocalized in three discrete cellular geometries. Asymmetric localization of GM130 and Pericentrin to one extreme of the nucleus in elongated cells was denoted Class I (Figure 5A″). Central positioning of GM130 and Pericentrin adjacent to or above the nucleus was denoted Class II (Figure 5B″ and 5C″). Class III included geometrically rounded cells with eccentric GM130 and Pericentrin (Figure 5D″). Finally, GM130 was occasionally observed as dispersed foci (not shown), Class IV, which is most likely the configuration in mitotic cells [48]. The tabulated results of several experiments are shown (Figure 5E). A similar frequency of wild-type Class I PGCs was observed in static and graded SCF (69% and 73%, respectively). This distribution of the Golgi and centrosome appears to be nonrandom given the relatively large cellular area occupied by the PGC nucleus. Strikingly, a significant overall reduction of Class I Golgi position was observed in *Ror2*: 45% in static SCF and 48% in a gradient, both of which differ from wild type (p = 0.005). This result suggests that the coordination of centrosome/Golgi position and cell shape is affected in *Ror2* PGCs. However, if we consider the Golgi position apart from cell shape—since the rounded Class III cells could retain molecular and organelle polarization– it becomes apparent that the cells in Class III also exhibit Golgi/centrosome asymmetry. In this line of reasoning, we find that the frequency of combined Class I and III PGCs does not differ between wild type and *Ror2* in graded SCF (p = 0.23) and is barely significant in static SCF (p = 0.04). This analysis could suggest that *Ror2* PGCs are defective in cell elongation, but not polarized positioning of the centrosome and Golgi. Conversely, if we compare only geometrically elongated cells, or those in Classes I and II, we find a decreased incidence of polarized Golgi position (Class I) of *Ror2* PGCs cultured in static SCF (p = 0.029), but not in gradient SCF (p = 0.18) compared to wild type. Given that the majority of *Ror2* PGCs elongate to some degree in SCF, this discrepancy in Golgi position could reveal a more subtle defect in their polarized response. Finally, although a rare class, the incidence of Class IV or dispersed GM130 appears elevated in *Ror2* PGCs (Figure 5E). This uptick could reflect a slight increase in proliferation of the mutant PGCs that we have observed in vitro (Figure S7). Taken together these experiments demonstrate a decoupling between cell elongation and polarized position of the Golgi and centrosome in *Ror2* mutant PGCs; however with the alternate interpretations of Class III cells as either randomly positioned or polarized Golgi/centrosome within a rounded cell, it remains possible that Ror2 acts as a cell polarity effector or else in the associated cell shape changes.

### ROR2 exhibits a polarized distribution in migratory PGCs in vivo and in vitro

**Figure 6:**
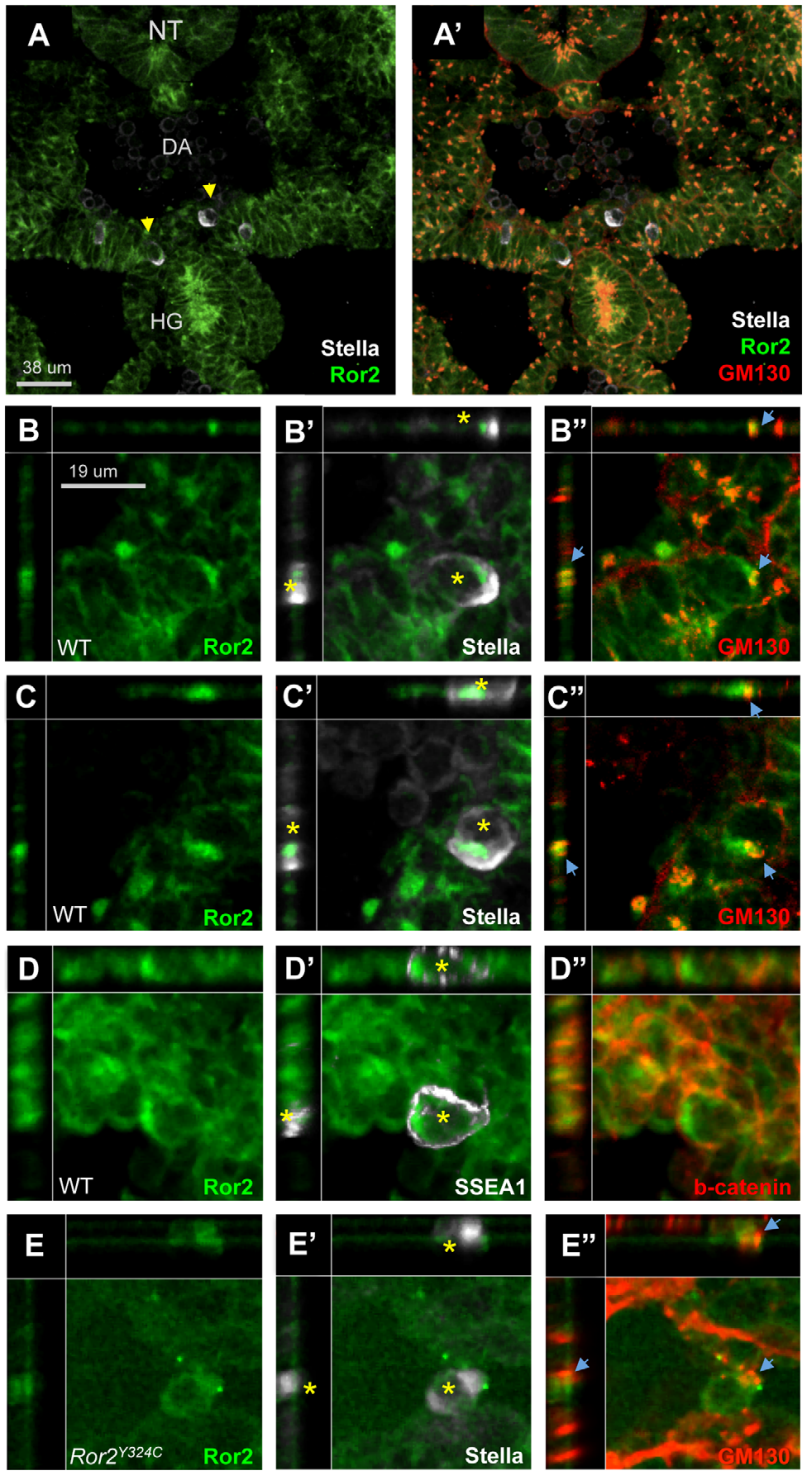
Asymmetric localization of ROR2 in polarized migratory PGCs in vivo. (A–E) Histologic sections from e10.25 WT embryos were immunostained with Stella, Ror2 and GM130 antibodies and PGCs migrating through the dorsal mesentery (A–A′) were examined (arrowheads). Viewed in the xy plane from above, as well as xz and yz cross sections (top and sides of panels) Ror2 and Stella distribution appeared asymmetric in most WT PGCs at this stage (asterisk, in B, B′, C, C′), and both signals were on the same side of the cell as GM130 (B″, C″). SSEA1 staining was not similarly asymmetric on PGCs (D, D′), nor was membrane-associated β-catenin (D″). On *Ror2^Y324C^* PGCs, Ror2 signal was very weak, and did not appear polarized, nor did Stella (E–E′), despite the presence of asymmetric GM130 (E″, arrowhead).

Several previous studies have implicated Ror2 in cell polarity, including polarized cell division in *C. elegans* [14], directional migration in the limb and several mammalian cell lines [18], [26], [49], and apicobasal polarity in the mouse gut [9]. A polarized distribution of ROR2 within the developing gut epithelium [9] prompted us to examine ROR2 localization in PGCs following culture in SCF. Immunofluorescence revealed asymmetry of ROR2 within the cytoplasm as well as on the surface membrane of PGCs. The distribution of ROR2 at one extreme of the cell coincided with GM130 (Figure 5F, 5F′). ROR2 was also observed prominently on the membrane protrusions of cultured PGCs. Returning to the embryo, we examined the subcellular distribution of ROR2 and GM130 in PGCs in vivo. In e10.25 histologic sections, immunostaining revealed an apical enrichment of ROR2 in the hindgut and dorsal neural tube, colocalized with GM130 (Figure 6A–6A′). At this stage, PGCs identified by the expression of Stella are migrating through the dorsal mesentery toward the gonadal ridges (Figure 6A). Within these PGCs, ROR2 appeared to be enriched on one side in most instances (Figure 6B, 6C, 6D). This enrichment was coincident with GM130 (Figure 6B″, 6C″) and, unexpectedly, Stella (Figure 6B′, 6C′). As a polarized Stella distribution has not been previously reported, we wondered whether this pattern could reflect the plane of section. When PGCs were instead immunostained with the SSEA-1 antibody, the asymmetric distribution of ROR2 persisted (Figure 6D), but SSEA-1 appeared to be localized consistently around the PGC border (Figure 6D′), as did β-catenin (Figure 6D″). Together, these data demonstrate a polarized distribution of ROR2 in PGCs that are responding to chemotactic cues in vitro and migrating in vivo. Its localization on filopodia and segregation on the same side of the cell as the Golgi suggests Ror2 could be important in the polarization response of the cell in response to SCF. Upon examining histologic sections from *Ror2^Y324C^* embryos, we did not observe a comparable degree of asymmetric ROR2 distribution or Stella distribution in PGCs, and GM130 staining was present but dimmer (Figure 6E, 6E′, 6E″). This result suggests that a functional Ror2 receptor could localize asymmetrically on a PGC responding to chemotactic cues in order to amplify or enhance the polarized response.

### Ror2 promotes elongation of migratory PGCs in vivo

**Figure 7:**
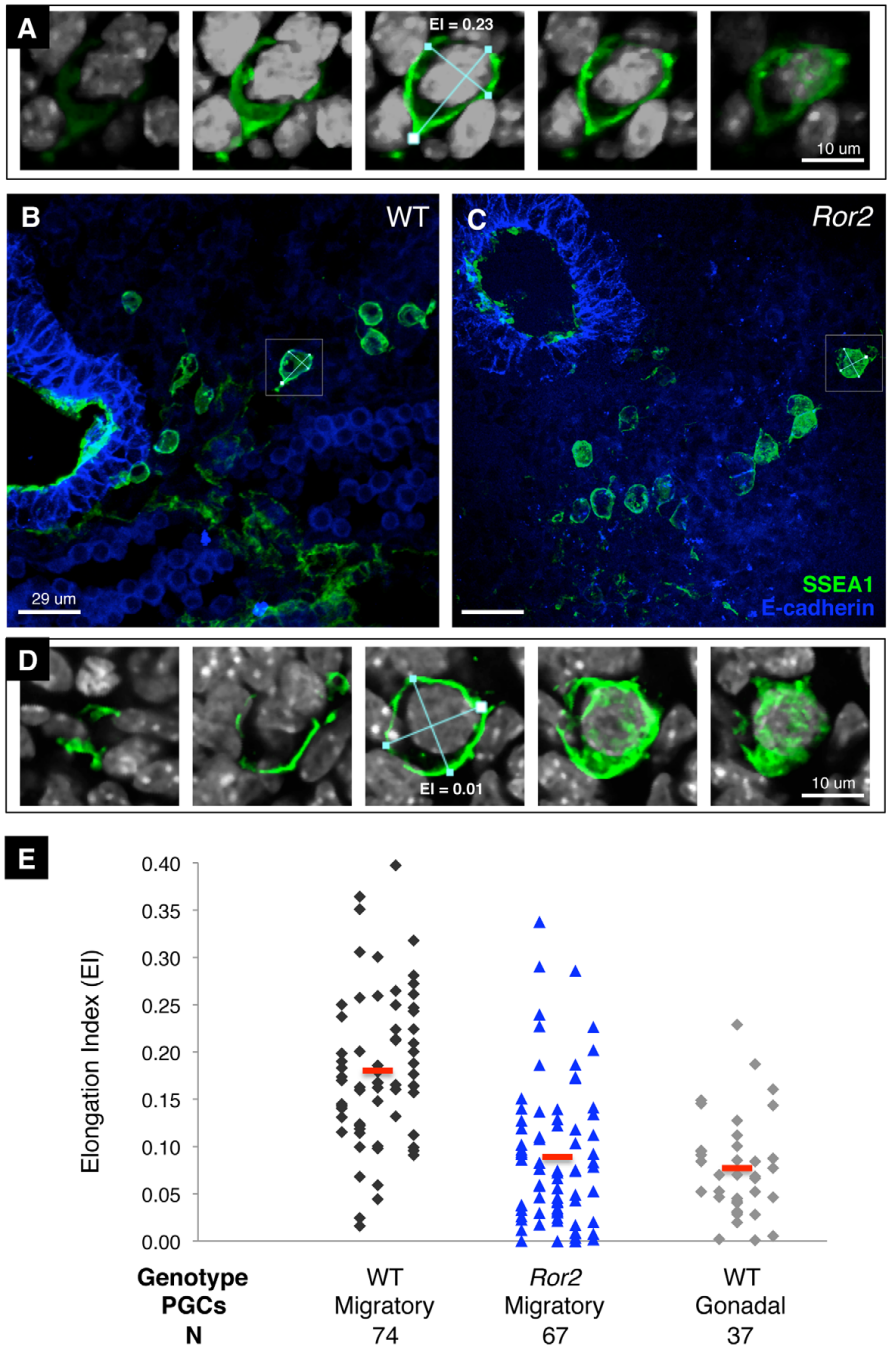
Reduced elongation of migratory *Ror2^Y324C^* PGCs in vivo. (A) WT migratory PGC stained with SSEA1 (green) and DAPI (grey) shown in five sections at different levels through a confocal stack. The axes are measured (blue lines) in the largest plane of the cell, shown in the middle panel, resulting in an EI = 0.23. (B) A confocal stack from a WT e10.25 embryo section stained with SSEA1 and E-cadherin to delineate the hindgut (left). Autofluorescent erythrocytes are present in the lower right quadrant. Filopodia and lamellopodia can be seen on many PGCs; the cell in (A) is boxed. (C) A similar histologic section from a *Ror2^Y324C^* embryo posterior, showing several SSEA1^+^ PGCs in the dorsal mesentery, many of which appear rounded. The boxed PGC is represented in (D), where measurement of the long and short axis in a plane yields an EI = 0.01. (E) Accumulated measurements for EIs of migratory WT and *Ror2* mutant PGCs from e9.75–10.75 histologic sections. Postmigratory WT e10.75 (“Gonadal”) PGCs are shown at right in grey and the mean EI in red.

We next asked whether the elongation phenotype of *Ror2* PGCs in culture could be observed in vivo. As the expression of KitL changes dynamically after 9.5 to become restricted to the genital ridges [2], PGCs migrating within the dorsal mesentery toward the gonadal ridges probably experience a gradient of secreted KITL analogous to the graded SCF in vitro. We examined PGCs migrating through the dorsal mesentery of sectioned e9.75–e10.75 embryos using DAPI and SSEA-1 staining to ensure that the entire cell was captured. Visually, many PGCs in *Ror2^Y324C^* embryos at this stage appeared rounded (Figure 7B, 7C). In confocal stacks, we located the largest cellular cross section for measuring the longest cellular axis and the short axis orthogonal to this one (Figure 7A, 7D). In migratory wild-type PGCs, we measured a mean EI of 0.18±0.08 (Figure 7E). This is in line with the mean EI of 0.20±0.12 that was determined in two dimensions from SCF-gradient cultured PGCs. In *Ror2^Y324C^* embryos, migratory PGCs exhibited a mean EI of 0.09±0.07, which is significantly less than in wild type (p<0.0001). For comparison, we examined PGCs located within the gonads of e10.75–11.5 embryos, since previous studies reported that following their arrival in the gonad, PGCs acquire a rounded morphology [50]. Wild-type postmigratory PGCs measured 0.08±0.05 mean EI. Together these in vivo observations suggest that Ror2 enhances the polarized response that leads to cell elongation of migratory PGCs.

## Discussion

The trafficking of PGCs through the embryo is a widely conserved process across many vertebrates and invertebrates [1]. Success is critical for fertility of the organism and propagation of its genome, and thus subject to selection. Failure has been linked to the development of germ cell tumors, because surviving mislocalized PGCs are subject to transformation [2], [51]. Our knowledge of the mechanisms underpinning PGC migration in mammals is limited [52]. Through forward genetics, we have implicated a new pathway in the migration of PGCs. Here we have identified an ENU allele of Ror2 that leads to a diminution of PGCs in the fetal gonads. Mutants in the putative ligand *Wnt5a* phenocopy *Ror2^Y324C^*, and both exhibit reduced efficiency gonad colonization by PGCs. In culture, we show that the growth factor SCF induces polarized alignment and cell shape change in wild-type PGCs that is depends on Ror2. Similar elongation and orientation phenotypes in *Ror2^Y324C^* suggest that Ror2 acts intrinsically in PGCs to enhance their polarized migration toward KITL.

### What is the primary defect in Ror2 mutant PGCs?

Our analysis shows a surge in apoptosis, an increase of ectopic PGCs in *Ror2^Y324C^* and *Wnt5a*, and disrupted hindgut architecture in *Ror2^Y324C^* embryos; consistent with this, PGC diminution in e11.5 gonads and concomitant increase in ectopic PGCs was reported in *Wnt5a* mutants as this manuscript was under revision [53]. However it remains unclear which of these three defects– apoptotic elimination, mismigration or anatomic barriers– primarily causes the PGC phenotype. On one hand, PGCs that migrate inefficiently could be increasingly subject to death, or on the other hand, dying or unhealthy PGCs could migrate poorly. This dilemma is resolved by rescuing cell death with genetic ablation of the proapoptotic gene *Bax*, which does not restore the loss of PGCs in *Ror2^Y324C^* gonads. By contrast, in *Steel* mutants, which lack both membrane and secreted KitL, one or two *Bax* null alleles is sufficient to rescue *KitL^Steel/Steel^* gonadal PGCs [2]. Together these results argue that migration is the primary defect in Ror2 PGCs, and apoptosis in the periphery arises as a consequence of reduced survival factor exposure. The possibility remains that ectopic *Ror2* PGCs are increasingly sensitive to the withdrawal of survival factors. KITL and SDF1, known to be the most important PGC survival factors [42], [43], [54], are both concentrated in the e11.5 gonads and their absence in peripheral tissues likely leads to *Ror2* PGC death.

### Does Wnt5a function as a chemoattractant in PGC migration?

In the developing mammalian palate, a series of bead and cell implantations suggest that WNT5A is sufficient for directional movement of cells via Ror2 [15]. However, similarly implanted beads coated with WNT5A did not divert the migration of PGCs in our studies (Figure S6), and we could not detect any role for Wnt5a as a chemoattractant. Instead, we suggest that Wnt5a may act permissively in PGC migration. This is not unprecedented, as in a melanoma cell line, Witze et al. showed that WNT5A acts permissively to regulate the polarized distribution of adhesion receptors in response to a chemokine gradient [26]. In PGCs, the known chemoattractive factors include SDF1 [42], [43] and KitL [2], [41], [55]. Recognized as the most critical growth and survival factor for PGC, KitL was first postulated as a guidance cue for PGCs from the analysis of the *Steel^Dickie^* mutant [56]; SCF (secreted KitL) was later shown to induce PGC migration ex vivo, as well as inducing cell shape changes [46], [57]. Aberrant cell shape was previously noted in PGCs from *Steel* mutants [58]. When the survival of *Steel* PGCs was restored in compound mutants with *Bax*, functions for KitL in motility, adhesion and colonization of the gonad were identified [2], [41]. The resemblance of these cellular phenotypes to what we observed in *Ror2^Y324C^* PGCs prompted us to ask whether Ror2 could enhance the response to KitL chemotactic cues.

### What is the role of Ror2 in PGC migration?

Our ex vivo experimental approach addresses three separate aspects of cell migration: polarity, cell shape change and orientation toward a chemotactic cue. We find that a gradient of SCF induces geometric elongation as well as a nonrandom alignment of wild type PGCs within the field. This chemotropic function of SCF was previously recognized in mast cells but not PGCs [59], [60]. Similar to a recent report [57], our results also reveal a chemokinetic, or non-directional function of SCF in migration, as wild-type PGCs assumed an elongated, polarized morphology when SCF was uniformly present. This morphology was accompanied by a polarization of the Golgi apparatus, which is typical of migratory cells [61]. In all of these cellular behaviors, *Ror2* PGCs exhibit a mitigated response to SCF; their orientation is randomized instead of aligned with respect to the gradient, they elongate less, and the frequency of polarized Golgi distribution reduced. We find, strikingly, a similar difference in the shape of migratory PGCs in *Ror2* mutant embryos. This result confirms in vivo a function for Ror2 in the polarized migration of PGCs. Taken together, the in vivo and in vitro experiments suggest that Ror2 signaling enhances the chemotactic response of PGCs to KitL emanating from the gonadal ridges. Although the precise function of Ror2 in this polarized migration remains to be determined, its nonrandom protein distribution throughout migratory PGCs may provide a clue. The observed pattern of ROR2 within the cytoplasm and near the cell membrane is reminiscent of the asymmetry within the hindgut epithelium [9]. The potential colocalization with the Golgi apparatus is intriguing and warrants further investigation. On the other hand, the distribution of ROR2 on membrane protrusions is reminiscent of the reported expression on the dendrites of hippocampal neurons [62]. The altered distribution of ROR2 in *Ror2^Y324C^* PGCs argues for its specificity and functional significance, and leads us to propose that ROR2 distribution becomes polarized in response to directional KitL cues and thus reinforces the polarization of the cell. In other words, Ror2 might potentiate asymmetry in Kit signaling, or even transform it from a general signal to a polarized signal. It is also possible that the localization of ROR2 on protrusions promotes the growth and selection of filopodia into a clear lamellopodia or a leading edge, similar to the axonal path finding function of the homolog in *C. elegans* [7]. Elucidating the dynamic distribution of ROR2 in PGCs undergoing polarized responses will be an important future pursuit.

### How does Ror2 enhance the response to KitL?

The connection established in these studies between Ror2 and SCF-induced cell polarization is new, and the molecular nature of the relationship is unclear. The robust evidence for the specificity of SCF for cKit rules out the possibility of biochemical interaction between SCF and Ror2 [63]. However, the absence of feeder cells or serum and purity of sorted PGCs in our culture system demonstrates that both proteins are acting in the same cell. Based on the detection of *Wnt5a* transcript and protein in PGCs, a plausible scenario would involve autocrine WNT5A secreted from PGCs in the cultures. In this model, PGC polarization is initiated by KitL and amplified by localized extracellular concentrations of WNT5A secreted from either PGCs or surrounding mesentery. In regions of high Wnt5a expression, which correspond to successive targets of PGCs such as the hindgut and the gonadal ridges, the sensitivity to KitL may be amplified to help guide PGCs toward these targets. Within PGCs, Ror2 engagement by Wnt5a could lead to the redistribution of ROR2 at the cell surface, for example through ligand-receptor endocytosis [64]; this could rapidly lead to asymmetric ROR2 distribution between leading and trailing edges of the cell. Ror2 could ultimately reinforce the polarization response initiated by KitL-cKit in a number of different ways, such as by augmenting common downstream signaling components, such as cytoskeletal reorganization machinery, or perhaps by inhibiting the responsiveness to KitL in some regions of the cell. Understanding the relationship between Wnt5a-Ror2 and KitL-cKit at a mechanistic level is an important next step. Both tyrosine kinase receptors function in the development of multiple tissues as well as cancer; cKit is a proto-oncogene implicated in melanoma [65] and gastrointestinal stromal tumors [66], and Wnt5a expression has been correlated with tumor invasiveness [25]. Therefore the relationship between these two pathways is likely to extend beyond PGCs.

## Materials and Methods

### Animals

All animal work was carried out in compliance with care and use standards at each institution. *Ror2^Y324C^* was identified in a recessive ENU screen at e9.5 for mouse mutants with PGC defects [23]. Other mouse strains used included: *Bax* (MGI:1857429), *Wnt5a* (MGI:1857617), and *Oct4-ΔPE-GFP* [67] with genotyping performed as described elsewhere [19], [50]. Mice were maintained on C3H or mixed C3H/FvB genetic backgrounds. Embryos were generated in timed matings by monitoring for copulatory plugs. Pregnant females were sacrificed and embryos staged by the following anatomic landmarks: 27–33 somite pairs was designated e10.0, 34–39 somite pairs as late e10, 45–48 somite pairs and the appearance of the otic vesicle as e11.5, and the presence of embryonic kidneys designated e12.5; gonad sex was determined by the appearance of tubules and the coelomic vessel in e12.5 males, and SRY genotyping [68].

### Mapping

MIT SSLP markers were used to map Ror2^Y324C^ to chromosome 13 to a ∼10 Mb interval between D13 MIT 176 and D13 MIT 13. Sequencing of the *Ror2* ORF revealed an A to G transition at position 1203, which creates a restriction site. *Y324C* genotyping was carried out by PCR amplification of a 238 bp fragment in 25 uL reactions heated to 95°C for 3 min, followed by 45 cycles of 94°C for 30 sec, 57°C for 1 min, 72°C for 30 sec, and a 7 min hold at 72°C using the following primers: 5′-ACC AGT GCT ACA ACG GCT CT-3′ and 5′-AGT TCC ACG CGT ACG TTT TT-3′). Subsequent digestion 5 h with 3 U HpyCh4 V (NEB) produced fragment sizes 152 and 86 bp for the wild type allele, 152, ∼50 and ∼30 bp for mutant allele.

### Flow cytometry and RT–PCR

Embryos were dissected at e9.5–11.5 in cold PBS/0.2% BSA and the posterior fragment or gonads dissociated in 0.25% trypsin/EDTA for 3–5 minutes at 37°C followed by 1 mg/mL DNaseI for 5 min. For Ror2 intracellular flow cytometry, cells were prepared using the Cytofix/cytoperm Kit (Beckton Dickson) and stained at 1∶50 (Santa Cruz Biotech A17). Live cell staining was carried out in phenol red-free DMEM/2% fetal bovine serum/10 mM EDTA. Dead cells were excluded on the basis of Sytox Blue (Invitrogen) signal. PGCs, delineated as Oct4(ΔPE)-GFP^+^ were sorted directly into lysis buffer and extracted with RNeasy Kit (Qiagen), DNAse I treated, and reverse-transcribed with qScript (Quanta Biosciences) or Superscript III (Invitrogen). PCR primers were designed with Primer Express software (Applied Biosystems). Amplification was carried out with 50 or 100 cell equivalents of cDNA on a Mastercycler EP (Eppendorf) using the following primer sets: 5′-GACTTCAACAGCAACTCCCAC-3′ and 5′-TCCACCACCCTGTTGCTGTA-3′ for *Gapdh*; 5′- AATGCACAACTGCCATCTCC-3′ and 5′-AGGAATGCCTAGACTACTGGAAAA-3′ for *KitL* [69]; 5′-AGTCTGGAGACCATGTTTCTGAAG T-3′ and 5′-TACTCTTCTCGTTGGGAATACTCAATA-3′ for *Oct4*; 5′- GAGATCAGCTTGTCCAC-3′ and 5′- AGCATCGCCTCTTGCCGG-3′ for *Ror2*, 5′- GCAGACCGAACGCTGTCATT-3′ and 5′- CCACAATCTCCGTGCACTTCT-3′ for *Wnt5a*.

### Immunostaining

For Western blotting, day 9.5 embryos were lysed in RIPA buffer containing 1% Nonident P-40, 0.25% Deoxycholate acid, 150 mM NaCl, 0.1% SDS, 50 mM HEPES (pH 7.4) and proteinase inhibitor cocktail (Roche). 40 ug protein was separated by SDS-PAGE gel and probed with anti-ROR-2 antibody (Santa Cruz Biotech, H-76).

For immunofluorescence histology, embryos fixed in 4% paraformaldehyde were embedded in OCT and cryosectioned at10 um. Slides were blocked 1 h in 10% calf serum + 0.1% Tween in PBS and stained overnight @ 4°C in the blocking buffer followed by 3×15 minute washes in PBS. Primary antibodies used included SSEA1 (Developmental Studies Hybridoma Bank, 1∶200), Wnt5a (R&D Systems AF645; 1∶20), activated Caspase 3 (Promega, G7481, 1∶250), phospho-histone H3 (Sigma, clone HTA28, 1∶200), E-cadherin (Invitrogen, 13–1900, 1∶200), Ror2 (Santa Cruz Biotech A-17 1∶50), Pericentrin (Covance, PRB-432C, 1∶50), GM130 (Beckton Dickinson, monoclonal, 1∶100), the latter was preceded by 2 min treatment in Ficin (Invitrogen). Bromodeoxyuridine (Abcam ab6326, 1∶40) was preceded by treatment in 4N HCl for 10 minutes and 5 min in 0.1 M Borate buffer pH 8.6. Secondary antibodies and fluorescent Phalloidin were purchased from Invitrogen were incubated for 1 hour at room temperature and used at 1∶200–1∶500. Sections were mounted in Vectashield (Vector Labs).

For whole mount immunofluorescence, embryos or gonads were fixed in methanol∶dimethylsulfoxide (4∶1) at −20°C overnight, rehydrated and rocked @ 4°C overnight in PBSMT (PBS/2% nonfat dry milk/0.5% Tween) with antibodies to SSEA1 (1∶200), cleaved PARP (Cell Signaling #9544, 1∶50),phospho histone H3 (1∶50), GCNA (a kind gift of George Enders, undiluted supernatant); triple washes in PBSMT were followed by overnight rocking with secondary antibodies diluted 1∶200 in PBSMT, followed again by washing 3×. Gonads were mounted on slides in Vectashield (Vector Labs), whereas whole embryos were serially dehydrated and cleared in Methyl Salicylate for viewing.

### PGC ex vivo culture

Oct4-ΔPE-GFP^+^ PGCs sorted from e9–9.75 embryos were seeded in chambered slides (Lab-Tek II) coated with 1 mg/ml Matrigel then incubated @ 37°C in 5% CO_2_ with DMEM/15% Knockout Serum Replacement (Invitrogen), 1000 U/mL LIF (Millipore), 5 uM Forskolin, and added 250 ng/mL WNT5a (RnD), 50 ng/ml SCF (Invitrogen). Gradients were produced by placing 100 ng/mL SCF in Matrigel along one edge of the chamber. Following culture, cells were fixed for 10 min in 4% paraformaldehyde and immunostained.

### Embryo culture

Embryos were dissected in RPMI/10 mM Hepes/10% FBS at e8.0 with membranes intact. Heparin coated glass beads (Sigma) or Affygel Blue 100–200 mesh beads (BioRad) were washed3× in PBS, soaked 1 hr in PBS-BSA, 25 ug/mL SCF (RnD), 25–50 ug/mL SDF1 (RnD), 50 ug/mL WNT5a (RnD) or 20–50-fold concentrated WNT5A conditioned media, washed, and implanted into the proximal allantois, hindgut pocket or axial mesoderm with microforceps. Embryos were cultured in organ culture dishes (Falcon) containing 50% DMEM HG with Pen-Strep/50% Heat inactivated Rat Serum (Taconic) at 37°C in 5% C0_2_ for 24 hours.

### Alkaline phosphatase activity

Fast red staining was carried out as detailed elsewhere [70].

### Image collection and analysis

Brightfield imaging was performed on an Olympus MVX10 stereomicroscope. Confocal imaging was carried out with a 10×, 20× or 63× objective on a Leica SP5 TCS microscope equipped with 405, 488, 543, 594, and 633 nm lasers. Stacks were analyzed using Volocity (Improvision).

The number of PGCs in wholemount e10.5 immunostained embryos or e11.5–12.5 gonads was estimated using a measurement protocol created in Volocity 5.0 acquisition software. Objects were identified by in the SSEA1 or GCNA channel using the “Find Objects Using Standard Deviation (SD) Intensity” task, with a lower limit of 3.2–3.7 SDs above the mean. Holes were filled in objects, and those under 20 mm^3^ were excluded, and touching objects separated using a size guide of 200 mm^3^ in the GCNA channel or 750 mm^3^ in the SSEA1 channel. “Exclude Objects by Size” task was repeated to eliminate objects less than 20 mm^3^ created by the previous command. Objects were visually inspected to determine the approximate size cutoff for single objects. For gonads colabeled with antibodies against PHH3 or cleaved PARP, subsequent selection of colocalized objects was carried out using the intensity and colocalization functions. All measurement results were exported to Excel (Microsoft) for calculations. Clustered objects exceeding the defined threshold of single PGCs were summed and divided by the average PGC size. For instances in which over 20% of the total measured GCNA volume remained clustered, the quantity of PGCs was estimated by dividing this total volume over the average object size in well scattered specimens (300 um^3^ used here for GCNA).

Cell axes lengths were quantified in confocal stacks visualized in Volocity using the measurement function and exported to Excel for analysis. Image reconstructions with the long axis marked were exported to ImageJ (NIH) for angle measurements.

## Supporting Information

Figure S1 PGCs are depleted in mutants homozygous for Ror2 targeted deletion allele. Gonads from age matched *Ror2^−/−^* and WT littermates on the C57Bl/6 background were stained in wholemount with GCNA antibody and PGCs quantified from confocal stacks. A significant depletion was observed in homozygous mutants (purple triangles) compared to WT (black diamonds) at e11.25 and e12.25 (A). At e11.5, compared to WT (B), *Ror2^−/−^* (C) gonads exhibit a skewed distribution of PGCs at the posterior (bottom), similar to *Ror2^Y324C^*.
(TIFF)

Figure S2 PGC proliferation and apoptosis in whole gonads. (A) Apoptosis and proliferation were simultaneously visualized in whole gonads, shown at e12.5 stained with GCNA (red), PHH3 (blue) and cleaved PARP (green) antibodies. PGCs were modeled as GCNA-delineated, size selected objects (B), and the subset of PHH3^+^ nuclei (C) and cPARP^+^ (D) objects were quantified (frequency of total GCNA^+^ objects indicated as percentage).
(TIFF)

Figure S3 Mitotic and dying PGCs can be identified in histological sections. Sagittal section through a WT e10.75 embryo stained with (A) SSEA1 (green), PHH3 (blue) and cPARP (red) antibodies reveals proliferating (arrowheads) and apoptotic (arrows) PGCs in the dorsal mesentery. Nuclear staining with DAPI (grey) confirms nuclear localization of PHH3 (B) and cPARP (C) signals, with the overlay of all channels shown in (D). Scale bar = 39 um.
(TIFF)

Figure S4 Posterior hindgut morphogenesis is abnormal in *Ror2* mutants. Hematoxylin and eosin staining of transverse sections of e9.5 wild-type and *Ror2^Y324C^* embryos, showing that the hindgut ring becomes increasingly dilated from upper trunk level (A) to lower trunk level (A′) and tail bud level (A″) in wild-type embryos (arrowheads). However, the hindgut ring was dilated at all equivalent positions in *Ror2* mutant embryos (arrows in B, B′, B″).
(TIFF)

Figure S5 PGCs are specified normally in *Ror2^Y324C^* and *Wnt5a* mutants. Embryos collected at ∼e7.5–8.0 were staged and alkaline phosphatase-stained PGCs were quantified. (A) *Ror2^Y324C^* did not affect the number of PGCs in heterozygotes (unfilled triangles) or homozygotes (blue triangles) compared to wild type littermates (black diamonds). Similarly, *Wnt5a* haploinsufficiency (unfilled circles) or ablation (pink circles) had no affect on PGCs at this stage (B).
(TIFF)

Figure S6 PGCs migrate inefficiently in *Ror2* and *Wnt5a*, but WNT5A is not a chemoattractant. (A–C) Embryos at e9.0 (12–14 somites) stained for alkaline phosphatase (AP) activity (red, arrowhead) reveal excessive ectopic PGCs throughout the allantois (B) and tail mesoderm (C) of *Ror2^Y324C^* (insets 2× magnification). (D–I) Beads impregnated with either BSA, SCF, SDF1 or WNT5A conditioned medium were implanted into e8.0 embryos before 24 hours culture and AP staining, and shown in ∼8 somite stage (D–F) and ∼12 somite stage embryos (G–H). PGC migration was not disrupted by the bead's presence (left) or altered by WNT5A-beads (E,H). By contrast, SDF1 and SCF were capable of diverting the migration of PGCs at close range (F,I red arrows)in n>3 embryos. (J) Culture of sorted e9.5 Oct4-ΔPE-GFP^+^ WT PGCs for 20 h on Matrigel in presence of Wnt5a (250 ng/ml) revealed a significant increase in PGC number (p = 0.007 by paired t-test).
(TIFF)

Figure S7 Increase in ex vivo *Ror2^Y324C^* PGC proliferation measured by BrdU incorporation. Unsorted PGCs mechanically dissociated from e9.5 embryos were cultured for 24 hours, with 1 hour in Bromodeoxyuridine (Brdu). After fixation, PGCs were identifiable by SSEA immunofluorescence (A) with 5 PGCs in the field (arrows). (B) Anti-BrdU staining shows incorporation by many of the feeder cells and one PGC (arrowhead), with overlaid channels in (C). Scale bar = 100 um. (D) Quantification of the % of PGCs that incorporate Brdu reveals a slight increase (p = 0.05) in *Ror2^Y324C^* compared to mixed WT and heterozygous PGCs.
(TIFF)
