# Supplementary figures and images for: Ror2 Enhances Polarity and Directional Migration of Primordial Germ Cells
Source: PLoS Genet. 2011 Dec 22;7(12):e1002428. doi: 10.1371/journal.pgen.1002428 (PMC3245308; doi:10.1371/journal.pgen.1002428)

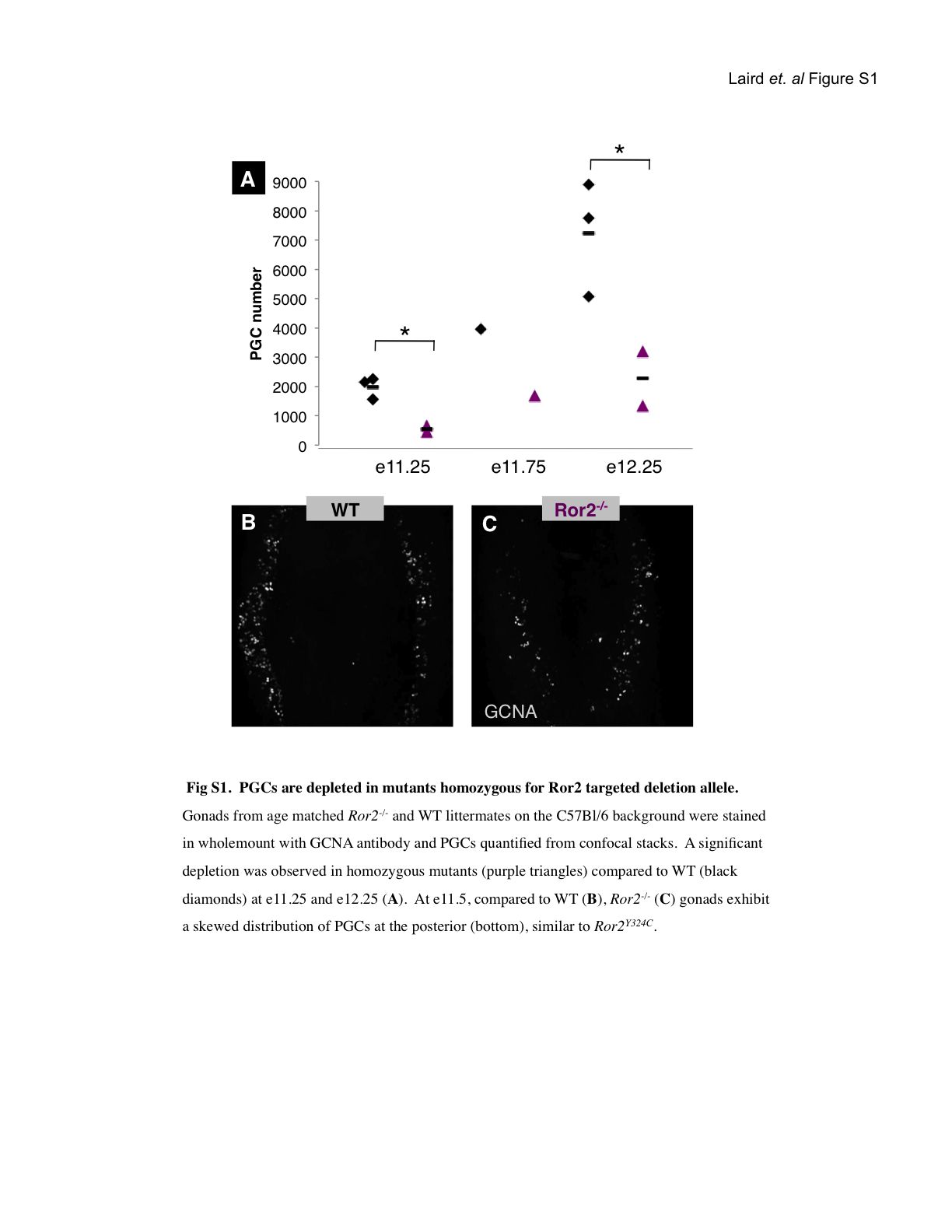

Supplement: Figure S1 — PGCs are depleted in mutants homozygous for Ror2 targeted deletion allele. Gonads from age matched Ror2−/− and WT littermates on the C57Bl/6 background were stained in wholemount with GCNA antibody and PGCs quantified from confocal stacks. A significant depletion was observed in homozygous mutants (purple triangles) compared to WT (black diamonds) at e11.25 and e12.25 (A). At e11.5, compared to WT (B), Ror2−/− (C) gonads exhibit a skewed distribution of PGCs at the posterior (bottom), similar to Ror2Y324C. (TIFF) [file pgen.1002428.s001.tif]

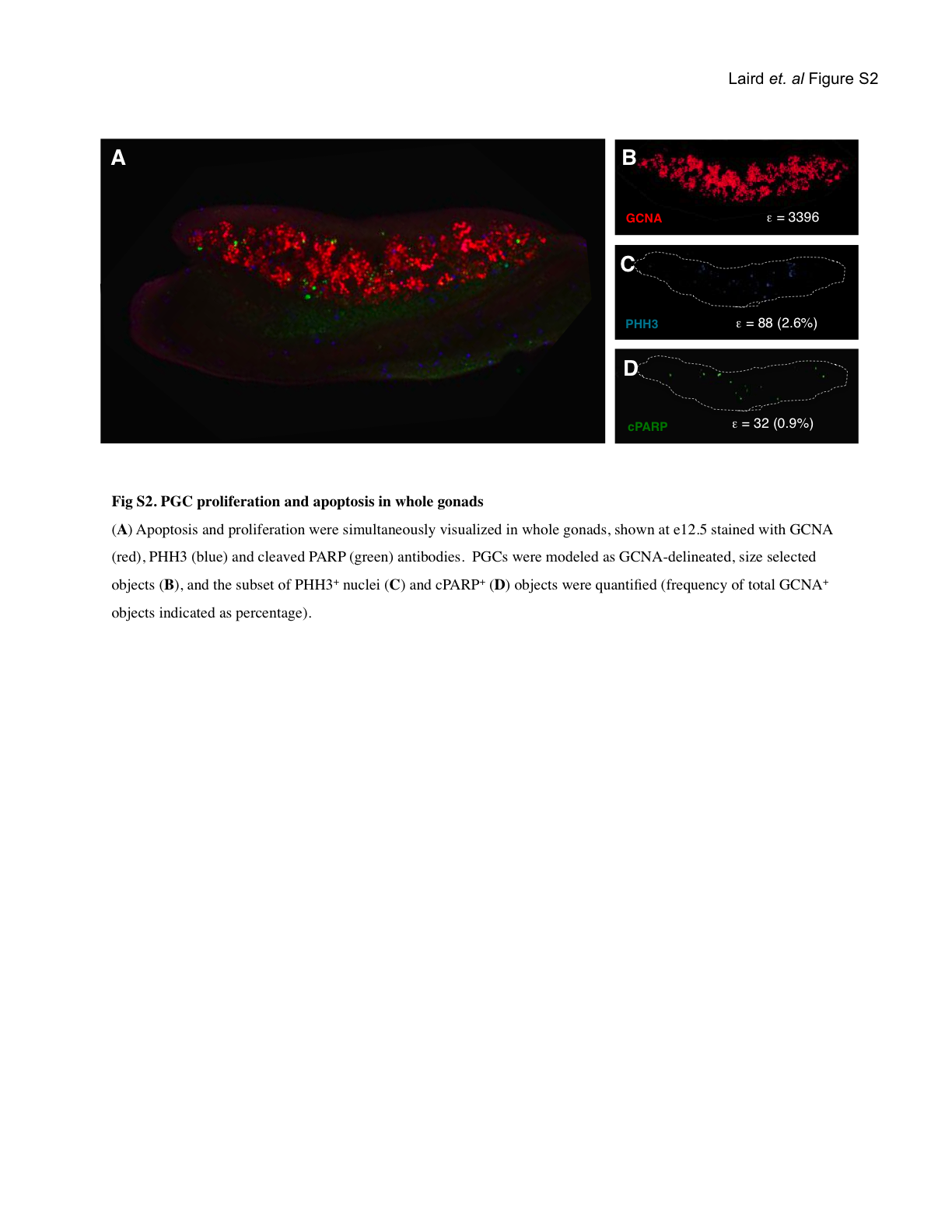

Supplement: Figure S2 — PGC proliferation and apoptosis in whole gonads. (A) Apoptosis and proliferation were simultaneously visualized in whole gonads, shown at e12.5 stained with GCNA (red), PHH3 (blue) and cleaved PARP (green) antibodies. PGCs were modeled as GCNA-delineated, size selected objects (B), and the subset of PHH3+ nuclei (C) and cPARP+ (D) objects were quantified (frequency of total GCNA+ objects indicated as percentage). (TIFF) [file pgen.1002428.s002.tif]

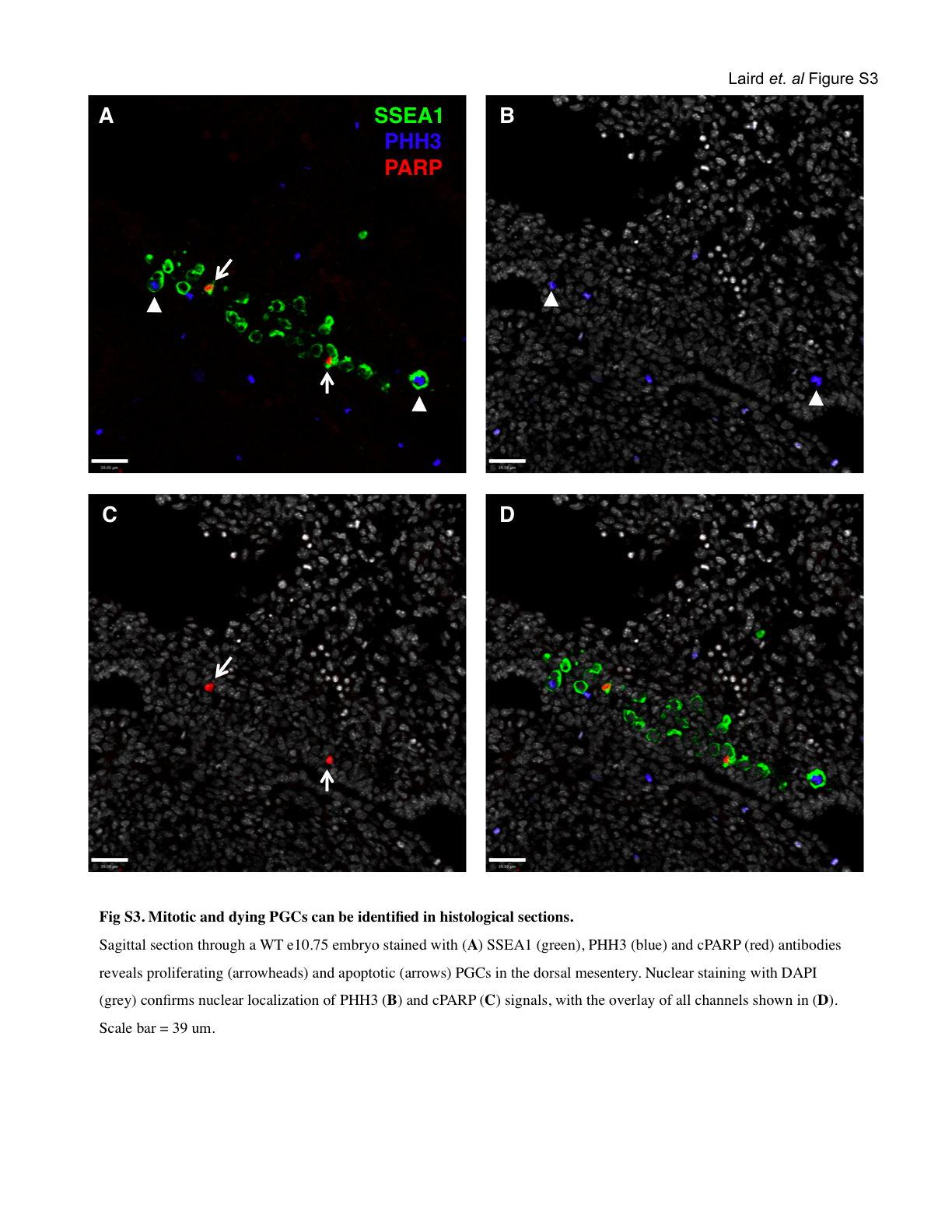

Supplement: Figure S3 — Mitotic and dying PGCs can be identified in histological sections. Sagittal section through a WT e10.75 embryo stained with (A) SSEA1 (green), PHH3 (blue) and cPARP (red) antibodies reveals proliferating (arrowheads) and apoptotic (arrows) PGCs in the dorsal mesentery. Nuclear staining with DAPI (grey) confirms nuclear localization of PHH3 (B) and cPARP (C) signals, with the overlay of all channels shown in (D). Scale bar = 39 um. (TIFF) [file pgen.1002428.s003.tif]

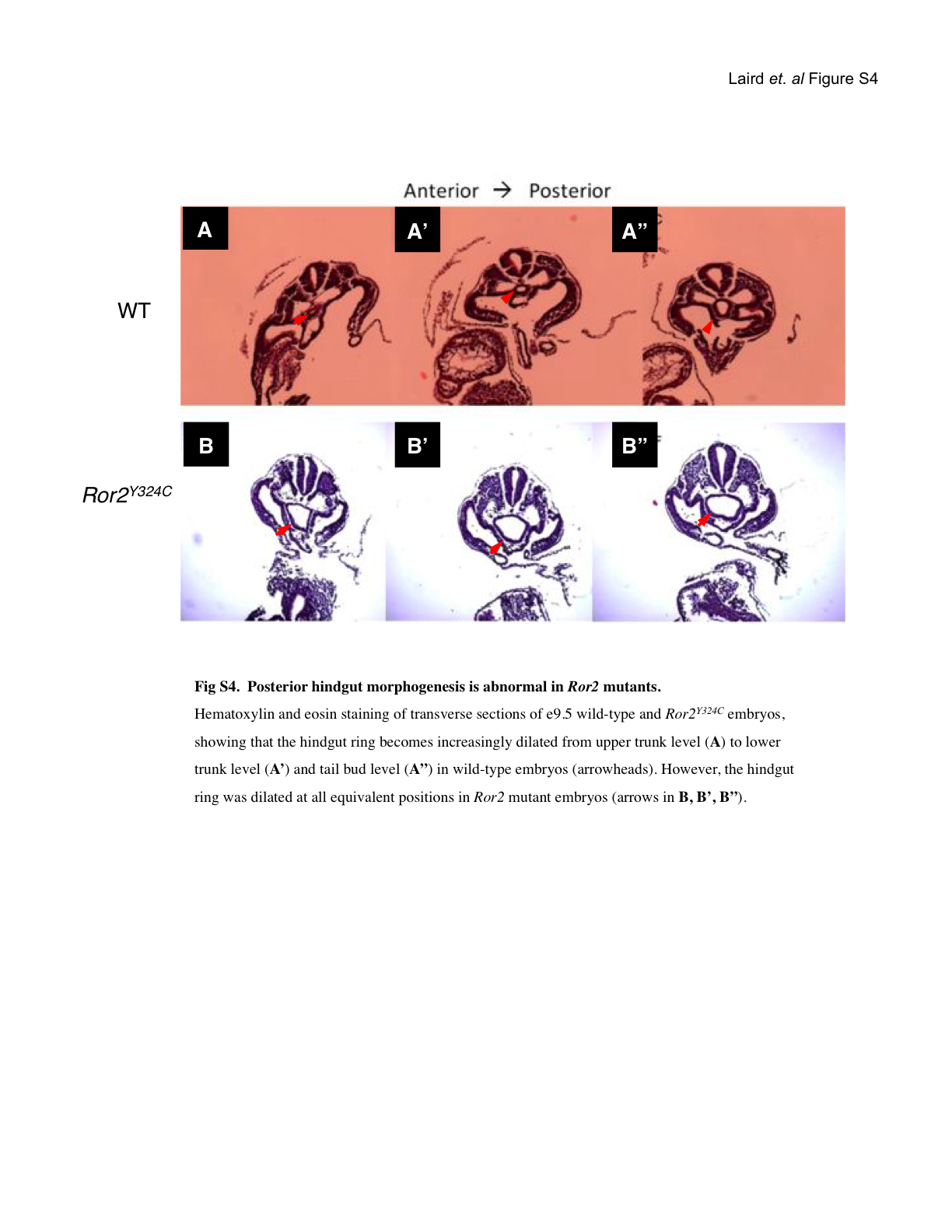

Supplement: Figure S4 — Posterior hindgut morphogenesis is abnormal in Ror2 mutants. Hematoxylin and eosin staining of transverse sections of e9.5 wild-type and Ror2Y324C embryos, showing that the hindgut ring becomes increasingly dilated from upper trunk level (A) to lower trunk level (A′) and tail bud level (A″) in wild-type embryos (arrowheads). However, the hindgut ring was dilated at all equivalent positions in Ror2 mutant embryos (arrows in B, B′, B″). (TIFF) [file pgen.1002428.s004.tif]

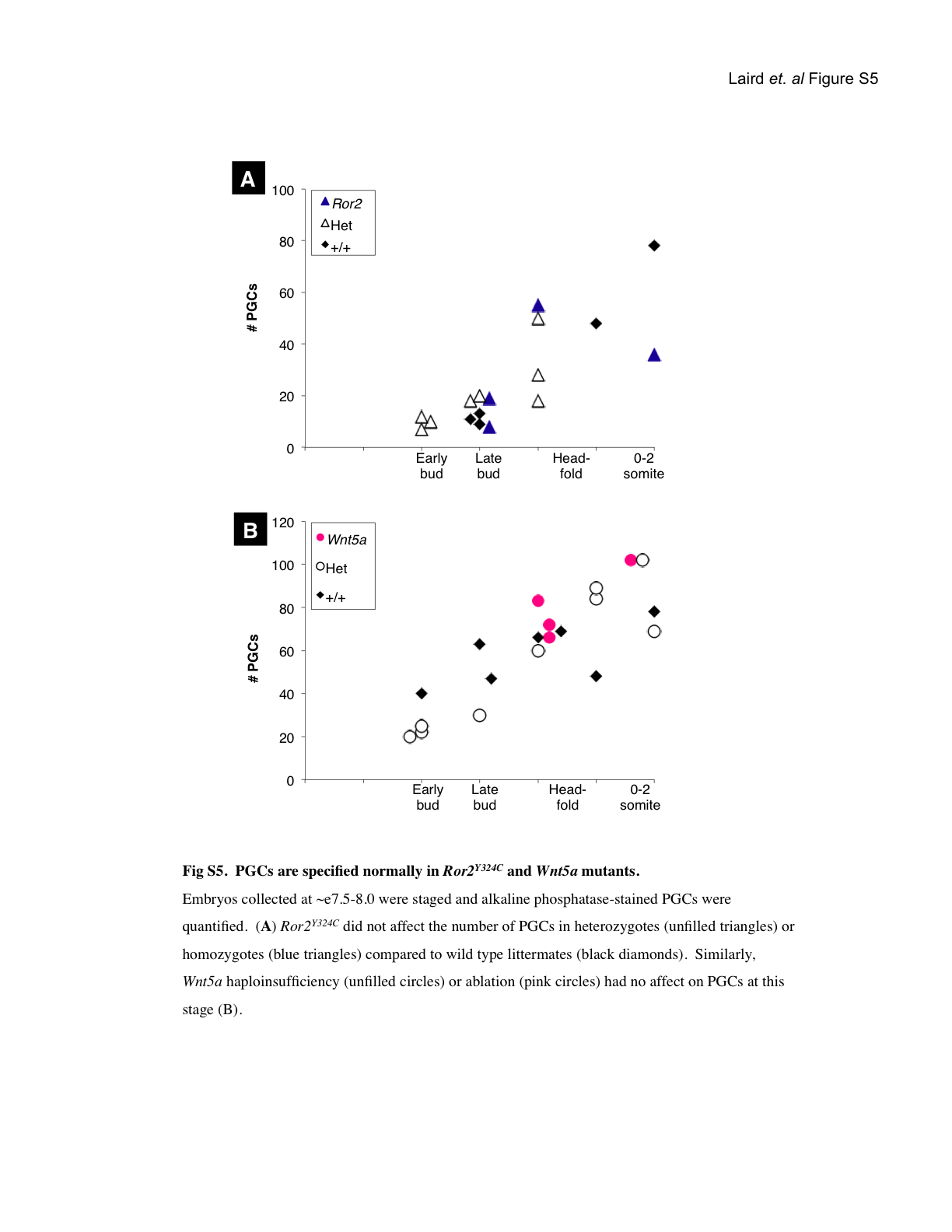

Supplement: Figure S5 — PGCs are specified normally in Ror2Y324C and Wnt5a mutants. Embryos collected at ∼e7.5–8.0 were staged and alkaline phosphatase-stained PGCs were quantified. (A) Ror2Y324C did not affect the number of PGCs in heterozygotes (unfilled triangles) or homozygotes (blue triangles) compared to wild type littermates (black diamonds). Similarly, Wnt5a haploinsufficiency (unfilled circles) or ablation (pink circles) had no affect on PGCs at this stage (B). (TIFF) [file pgen.1002428.s005.tif]

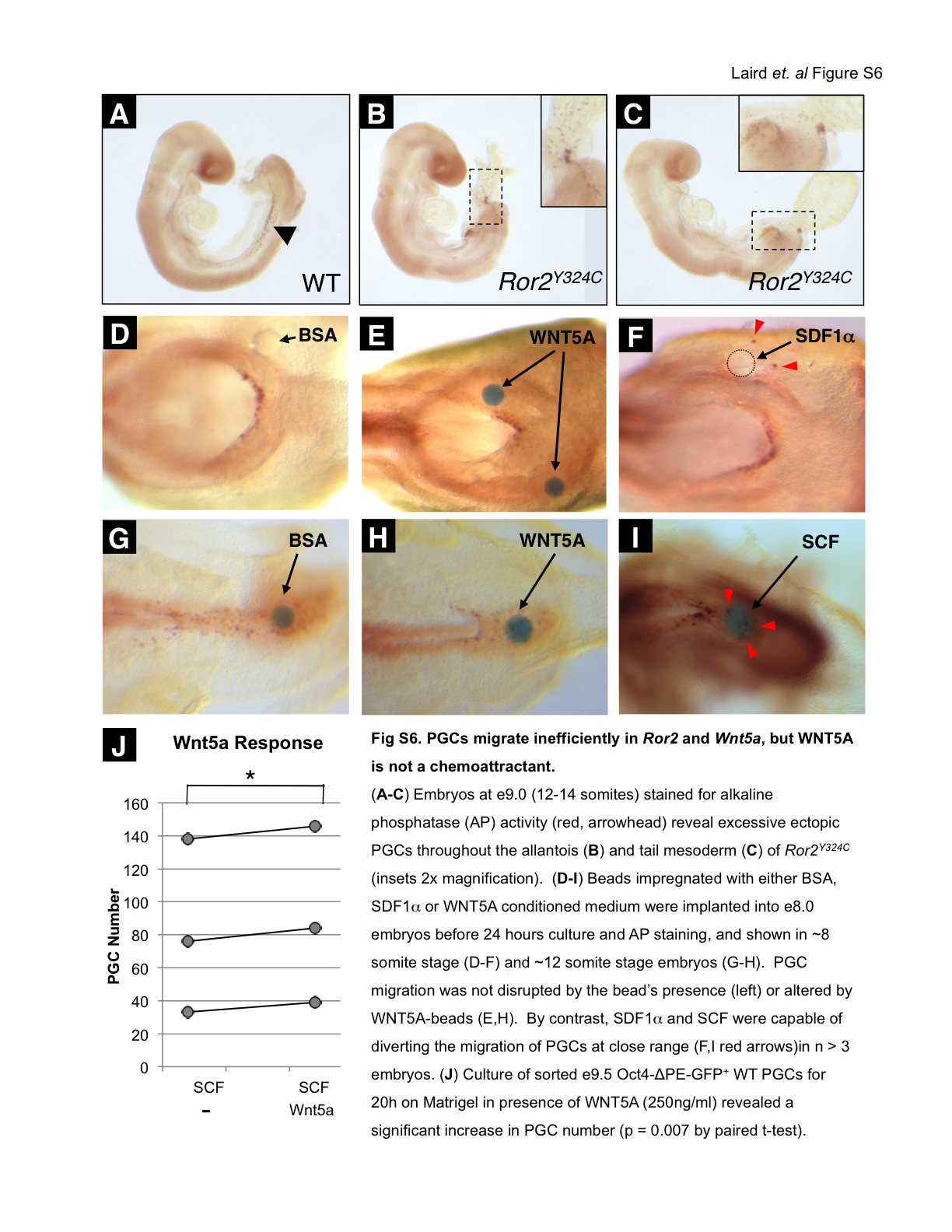

Supplement: Figure S6 — PGCs migrate inefficiently in Ror2 and Wnt5a, but WNT5A is not a chemoattractant. (A–C) Embryos at e9.0 (12–14 somites) stained for alkaline phosphatase (AP) activity (red, arrowhead) reveal excessive ectopic PGCs throughout the allantois (B) and tail mesoderm (C) of Ror2Y324C (insets 2× magnification). (D–I) Beads impregnated with either BSA, SCF, SDF1 or WNT5A conditioned medium were implanted into e8.0 embryos before 24 hours culture and AP staining, and shown in ∼8 somite stage (D–F) and ∼12 somite stage embryos (G–H). PGC migration was not disrupted by the bead's presence (left) or altered by WNT5A-beads (E,H). By contrast, SDF1 and SCF were capable of diverting the migration of PGCs at close range (F,I red arrows)in n>3 embryos. (J) Culture of sorted e9.5 Oct4-ΔPE-GFP+ WT PGCs for 20 h on Matrigel in presence of Wnt5a (250 ng/ml) revealed a significant increase in PGC number (p = 0.007 by paired t-test). (TIFF) [file pgen.1002428.s006.tif]

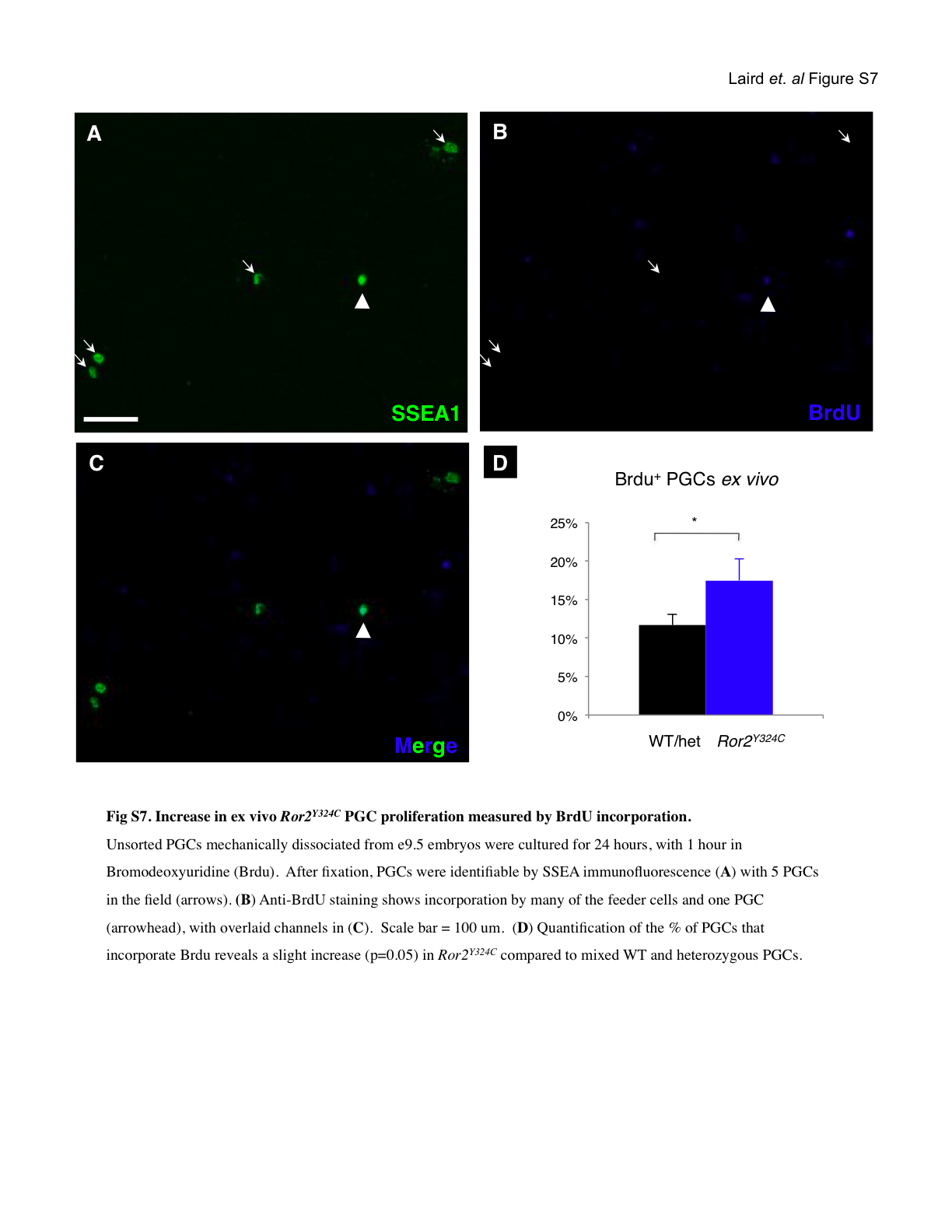

Supplement: Figure S7 — Increase in ex vivo Ror2Y324C PGC proliferation measured by BrdU incorporation. Unsorted PGCs mechanically dissociated from e9.5 embryos were cultured for 24 hours, with 1 hour in Bromodeoxyuridine (Brdu). After fixation, PGCs were identifiable by SSEA immunofluorescence (A) with 5 PGCs in the field (arrows). (B) Anti-BrdU staining shows incorporation by many of the feeder cells and one PGC (arrowhead), with overlaid channels in (C). Scale bar = 100 um. (D) Quantification of the % of PGCs that incorporate Brdu reveals a slight increase (p = 0.05) in Ror2Y324C compared to mixed WT and heterozygous PGCs. (TIFF) [file pgen.1002428.s007.tif]
